# Sexual Dimorphism in Cardiometabolic Diseases: From Development to Senescence and Therapeutic Approaches

**DOI:** 10.3390/cells14060467

**Published:** 2025-03-20

**Authors:** Thea Chevalley, Marion Dübi, Laurent Fumeaux, Maria Serena Merli, Alexandre Sarre, Natacha Schaer, Umberto Simeoni, Catherine Yzydorczyk

**Affiliations:** Developmental Origins of Health and Disease (DOHaD) Laboratory, Division of Pediatrics, Department Woman-Mother-Child, Lausanne University Hospital, University of Lausanne, 1011 Lausanne, Switzerland; thea.chevalley@chuv.ch (T.C.); marion.dubi@unil.ch (M.D.); laurent.fumeaux@gmail.com (L.F.); mariaserena.merli@irb.usi.ch (M.S.M.); alexandre.sarre@chuv.ch (A.S.); natacha.schaer@gmail.com (N.S.);

**Keywords:** cardiovascular disease, metabolic disease, developmental origins of health and disease, sexual dimorphism, cardiovascular therapies

## Abstract

The global incidence and prevalence of cardiometabolic disorders have risen significantly in recent years. Although lifestyle choices in adulthood play a crucial role in the development of these conditions, it is well established that events occurring early in life can have an important effect. Recent research on cardiometabolic diseases has highlighted the influence of sexual dimorphism on risk factors, underlying mechanisms, and response to therapies. In this narrative review, we summarize the current understanding of sexual dimorphism in cardiovascular and metabolic diseases in the general population and within the framework of the Developmental Origins of Health and Disease (DOHaD) concept. We explore key risk factors and mechanisms, including the influence of genetic and epigenetic factors, placental and embryonic development, maternal nutrition, sex hormones, energy metabolism, microbiota, oxidative stress, cell death, inflammation, endothelial dysfunction, circadian rhythm, and lifestyle factors. Finally, we discuss some of the main therapeutic approaches, responses to which may be influenced by sexual dimorphism, such as antihypertensive and cardiovascular treatments, oxidative stress management, nutrition, cell therapies, and hormone replacement therapy.

## 1. Introduction

Women represent half the population but are under-represented in medical research and drug development, leading to sex-based disparities in healthcare. Historically, there has been limited clinical research in women, possibly due to the greater susceptibility of men to develop chronic diseases compared to women in the same age group before menopause [1]. In studies using animal models, females have been under-represented because of their supposedly greater variability caused by the phases of their hormone cycles, making it more challenging to interpret results in females than in males. However, recent studies have shown, in contrast to general belief, that the variability in female rodents is no more consequential than that in males [2]. It is increasingly recognized that the burden, incidence, impact, symptoms, and treatment mechanisms of illnesses and health disorders differ between sexes [1]. Hence, it is imperative to ensure equal representation of females in studies to achieve more realistic results for clinical translation [3]. Preclinical and clinical studies have begun to address this issue and are providing evidence to help reduce the major gaps in our knowledge regarding the sex-specific biological responses observed in both health and disease, including in neurodegenerative and mental disorders, immune-related diseases, oncologic conditions, and cardiovascular morbidity. Nevertheless, despite these attempts, barriers still limit the inclusion of women in studies of therapeutic interventions, particularly when of reproductive age, pregnant, or lactating, and alternative clinical trial designs are needed for these groups [1].

It is increasingly recognized that sex and gender have a combined influence on cardiovascular and metabolic outcomes, impacting disease management. Differentiating between sex and gender is crucial: sex refers to genetic and biological aspects, whereas gender encompasses sociocultural roles, perception and expression of identity, and behaviour [4]. Western healthcare systems often overlook sociocultural factors in cardiometabolic management. Some socioeconomic factors, such as low income and limited healthcare access, are more common in women, complicating comparisons among patients with cardiovascular and metabolic disorders [5].

In this narrative review, we examine the impact of sexual dimorphism due to sex on the development and presentation of cardiometabolic disease in the general population and in the context of the developmental origins of health and disease (DOHaD). We also explore some therapeutic approaches potentially affected by sex-related differences.

## 2. Cardiovascular Disease

Cardiovascular health refers to the normal functioning of the heart and blood vessels. Dysfunction of these organs leads to cardiovascular disease (CVD) (a global term encompassing several entities—notably, heart failure (HF), atherosclerosis, arterial hypertension, and stroke), which represents the leading cause of death worldwide. Several factors can contribute to CVD, including elevated blood pressure (BP) levels, high blood cholesterol, diabetes, and tobacco use. Genetic and developmental issues can also play a role. It is well established that CVD is one of many medical conditions that show sex-related differences. Cellular processes, including rhythmicity, lipid metabolism, fibrosis, and regenerative capacity, are known to differ in healthy male and female cardiac cells [6,7]. Thus, sex can affect the epidemiology, prevalence, clinical manifestations, progression, and outcome of CVD, as well as the effectiveness and possible adverse effects of medical therapies [1].

### 2.1. Sexual Dimorphism in Cardiovascular Health

Sex-related disparities in cardiac structure and function are observed in healthy adults of both humans and rodent species [8]. Men have larger hearts than women, with stronger contractile function; a man’s left ventricle is ~30% larger than a woman’s and his heart rate is ~5–10 beats per minute lower, contributing to a larger cardiac reserve [9]. Under physiological conditions, women have stronger diastolic function, and their resting heart rate is higher than that in men, with a longer QT interval and a shorter recovery time of the sinus node [10,11]. Therefore, women have exhibit greater age-related increase in ejection fraction than men [12]. In addition, during pregnancy, when the female cardiovascular system must adapt to the circulatory demands of the developing foetus, the mechanisms underlying autonomic control of vascular resistance and cardiac function, along with volume regulation and vascular/cardiac remodeling, differ from those in males [13].

### 2.2. Sexual Dimorphism in Cardiovascular Disease

**Figure 1:**
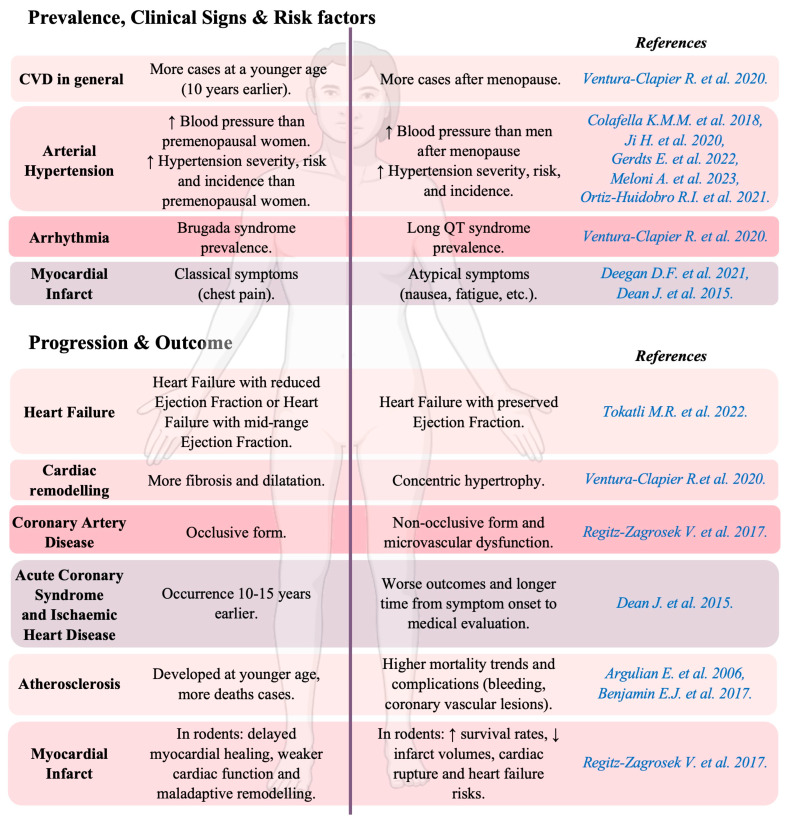
Sexual dimorphism in human cardiovascular disease. Figure comparing differences in prevalence, clinical signs, risk factors, progression, and outcomes of different cardiovascular diseases (CVDs) in women and men [3,8,9,14,15,16,17,18,19,20,21]. Created using a licensed version of BioRender.com.

Under pathological conditions, sex-related cardiovascular dimorphism is heightened, notably in terms of CVD prevalence, risk factors, clinical signs and symptoms, disease evolution, and outcome (Figure 1).

#### 2.2.1. Prevalence, Clinical Signs, and Risk Factors

In women and men, CVD follows different patterns; while incidence is lower and delayed by about 10 years in women, they face higher mortality and worse outcomes after acute events. In men, CVD risk increases steadily with age, whereas in women, it rises sharply after menopause [14].

Arrhythmia prevalence differs by sex: Brugada syndrome is more common in men (1:9), while long QT syndrome is more frequent in women (3:1). Though atrial fibrillation (AF) is less common in women, it carries a higher CVD and mortality risk, making female sex an independent risk factor in the CHA2DS2-VASc score [14,22].

Symptoms also vary: women with myocardial infarction (MI) are less likely to report chest pain and more likely to experience nausea, fatigue, dyspnea, or atypical signs like mandibular discomfort [8]. When angiography shows no obstructive CAD, their symptoms may be dismissed as “atypical”, despite risks from coronary microvascular dysfunction [15]. Arterial hypertension (≥130/85 mmHg) is a major HF and MI risk factor. BP levels are similar in childhood but diverge after puberty; by 18, men have SBP 10 mmHg higher than women. Pre-menopause, women have lower hypertension risk, but post menopause, their SBP rises more sharply, leading to higher hypertension prevalence [9,16,17,18,19]. Animal models also show sex-based variations in CVD progression, with male rats displaying greater cardiac dysfunction and hypertension severity. Salt-sensitive male Dahl rats are more likely to develop cardiac hypertrophy and left ventricular dysfunction than female rats [23]. In the spontaneous hypertensive rat model, there are sex differences in several aspects of cardiovascular function, including the severity of arterial hypertension. Both sexes are severely hypertensive, with SBP values ranging between 120 and 150 mmHg during the first 5 to 6 weeks of life [24]. However, from 8 to 20 weeks of age, the increase in BP is generally more rapid and significantly greater in males than in females [24]. In a rat model of intrauterine growth restriction (IUGR), Simoncini et al. reported an increased SBP only in 6-month-old male animals [25].

#### 2.2.2. Progression and Outcome

The progression and severity of CVD, as well as its outcome, can differ considerably between sexes. In HF, men more often have a reduced/mid-range ejection fraction, while women more commonly have a preserved ejection fraction [1]. Women show concentric hypertrophy, whereas men exhibit more fibrosis and dilatation [14]. Furthermore, men are prone to occlusive CAD, while women develop non-occlusive CAD and microvascular dysfunction [3]. Ischaemic heart disease and acute coronary syndrome (ACS) occur 10–15 years later in women but result in worse outcomes, including longer delays in seeking care and higher risk-adjusted bleeding from antithrombotic therapy [15,26]. Atherosclerosis is more common in men, who develop it earlier (40–60 years); however, women with the disease have higher mortality and more complications [20,21].

Animal studies have reported higher survival rates in female rodents than in males in the initial days following MI when subjected to ischaemic stress. Females have smaller infarct volumes and reduced rates of cardiac rupture within the first 5 days after MI. Additionally, females have better outcomes and a lower risk of developing HF, whereas males experience delayed myocardial healing, weaker cardiac function, and maladaptive remodeling [3].

## 3. Metabolic Syndrome

**Figure 2:**
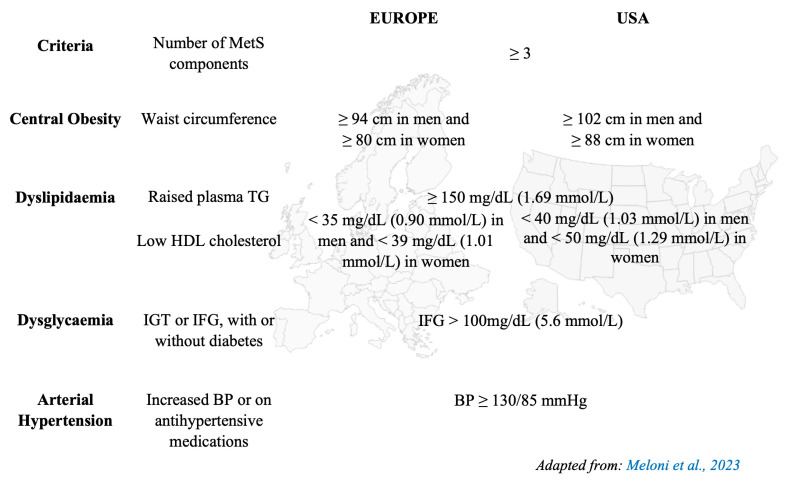
General criteria for the diagnosis of metabolic syndrome (MetS). Figure comparing criteria in Europe and America and between men and women for the diagnosis of metabolic syndrome. Relevant criteria include blood pressure (BP); impaired fasting glucose (IFG); impaired glucose tolerance (IGT); and levels of high-density lipoprotein (HDL), cholesterol, and plasma triglycerides (TGs) [18]. Created using a licensed version of BioRender.com.

Metabolic syndrome (MetS), or “syndrome X”, is characterized by a cluster of interconnected metabolic and cardiovascular risk factors, including central obesity (measured by waist circumference), insulin resistance (IR), dyslipidaemia, dysglycaemia, and arterial hypertension. The diagnosis of MetS requires the presence of at least three of these factors, each of which has sex-specific thresholds (Figure 2). Recently, additional components, such as non-alcoholic fatty liver disease (NAFLD) and obstructive sleep apnoea (OSA), have been included in the definition of MetS [18].

**Figure 3:**
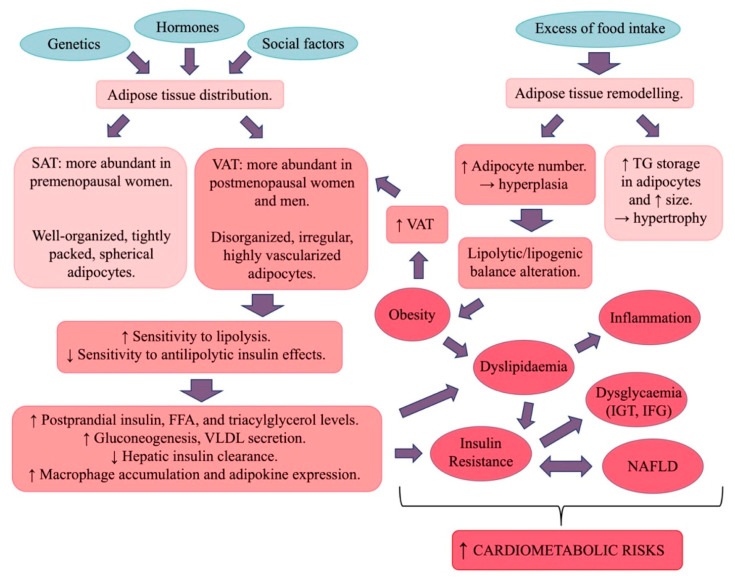
Connections between components of metabolic syndrome and risk of cardiovascular disease. Genetics, hormones, social factors, and food intake influence adipose tissue disposition. Fat accumulation leads to the development of several risk factors for cardiometabolic diseases. Abbreviations: FFA, free fatty acid; IFG, impaired fasting glucose; IGT, impaired glucose tolerance; NAFLD, non-alcoholic fatty liver disease; SAT, subcutaneous adipose tissue; TG, triglyceride; VAT visceral adipose tissue; VLDL, very low-density lipoprotein.

**Figure 4:**
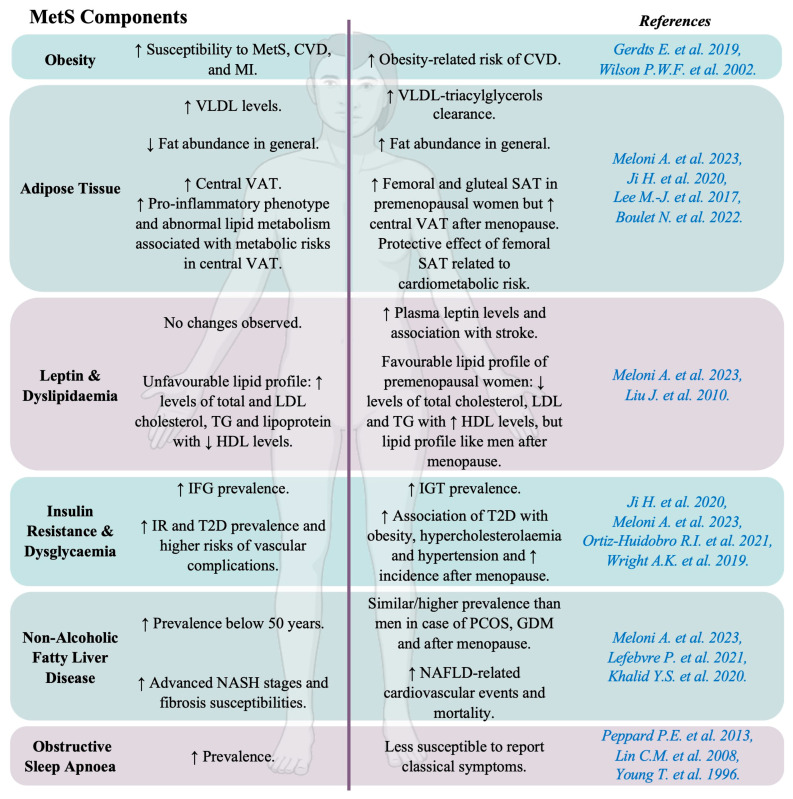
Sexual dimorphism in human metabolic syndrome (MetS) components. Figure comparing the difference in prevalence and consequences of several MetS components between men and women [16,18,19,28,29,30,31,32,33,34,35,36,37,38]. Abbreviations: CVD, cardiovascular disease; H/L/VLDL, high/low/very low-density lipFoprotein; IGT, impaired glucose tolerance; IFG, impaired fasting glucose; IR, insulin resistance; GDM, gestational diabetes mellitus; NAFLD, non-alcoholic fatty liver disease; (NASH) non-alcoholic steatohepatitis; PCOS, polycystic ovary syndrome; SAT, subcutaneous adipose tissue; T2D, type 2 diabetes; TG, triglyceride; (VAT) vascular adipose tissue. Created using a licensed version of BioRender.com.

MetS is independently associated with increases in CVD incidence and mortality, even after adjusting for potential confounders (Figure 3). It includes metabolic and haemodynamic risk factors contributing to atherosclerosis, CHD, and stroke [18]. Key contributors to MetS include hypercaloric diets, sedentary lifestyles, and genetic/environmental influences. Its prevalence depends on the age, race, geography, education, and income of the studied population [18]. Sex differences exist, with men facing a higher risk of MetS than premenopausal women [27] (Figure 4).

### 3.1. Obesity and Dyslipidaemia

#### 3.1.1. Obesity

Obesity was first recognized as a disease by the World Health Organization (WHO) in 1948, and today, this United Nations agency considers obesity to be a “complex chronic disease” [39]. Men and women are considered obese if they have a body mass index (BMI) ≥30 kg/m^2^ [40]. However, recent recommendations suggest that BMI-based definitions of obesity can both underestimate and overestimate adiposity, leading to misdiagnoses. BMI does not distinguish between fat and lean mass and does not take into account the distribution of fat. In addition, it provides no information on tissue and organ function, nor on an individual’s ability to perform daily activities, which are two essential criteria for assessing disease. Therefore, the use of other measurements besides BMI has been proposed to define obesity status, such as waist circumference, waist–hip ratio, or waist–height ratio [39].

Obesity significantly increases the risk of various diseases, including type 2 diabetes (T2D), CVD, and NAFLD. Notably, there are significant sex differences in adipose tissue distribution and obesity-related health risks [41]. Obese men are generally more susceptible to MetS, CVD, and MI than obese women [28]. However, findings from the Framingham Heart Study indicate a higher obesity-related cardiovascular risk in women (64%) compared to men (46%) [29]. In animal models, male mice on a high-fat diet (HFD) tend to gain more weight and exhibit a greater risk of IR than females [42].

#### 3.1.2. Adipose Tissue

Early studies on adipose tissue primarily focused on its role in energy storage. However, it is now recognized as a complex and dynamic organ composed of diverse cell types—including adipocytes, immune cells, endothelial cells, and blood cells—that collectively contribute to metabolic health [43].

Adipose tissue regulates fat storage and releases free fatty acids (FFAs) in an insulin-dependent manner. Adipocytes secrete lipoprotein lipase, which integrates into the plasma membrane of endothelial cells to hydrolyse triacylglycerols from chylomicrons and very low-density lipoproteins (VLDL), producing FFAs that are subsequently re-esterified into triacylglycerols by adipocytes. While men typically have higher VLDL levels, women clear VLDL-triacylglycerols more efficiently. During fasting, women exhibit greater lipolysis and FFA release, along with higher fatty acid oxidation during exercise [19].

Fat distribution differs between sexes: women generally have a higher total body fat percentage, with fat stored primarily in femoral and gluteal subcutaneous adipose tissue (SAT), while men tend to accumulate fat in central visceral adipose tissue (VAT) [30]. This sexual dimorphism becomes evident at puberty, with girls experiencing a rapid increase in total fat mass. By early adulthood, women typically have about 10% more body fat than men with the same BMI [31].

SAT is made up of tightly packed adipocytes, whereas VAT is more vascularized and contains disorganized adipocytes that are more sensitive to catecholamine-induced lipolysis and resistant to insulin’s antilipolytic effects. As a result, VAT contributes to increased FFA delivery to the liver, promoting gluconeogenesis and VLDL secretion. Higher lipolytic activity in visceral fat contributes to increased risks of dyslipidaemia and IR. Therefore, in men, central fat deposition is linked to a disturbance in fatty acid metabolism and a pro-inflammatory phenotype. Despite greater lipogenesis, female adipocytes are smaller, exhibit a higher lipolytic rate, and undergo greater metabolic turnover, leading to reduced visceral fat accumulation compared to men. In women, a greater gluteal–femoral fat mass is linked to better glucose and lipid metabolism and favourable adipokine and inflammatory profiles. However, after menopause, the differences in SAT and VAT diminish, leading to increased central adiposity and associated metabolic risks [18,19].

Similar sex-specific differences are observed in small animal models. Female mice exhibit adipose stem cell differences linked to genes involved in oestrogen signalling, homeobox transcription factor expression, and the renin–angiotensin–aldosterone system. Conversely, male mice are more prone to expanding visceral white adipose tissue, which is associated with a higher risk of T2D and CVD [44]. Additionally, female mice demonstrate greater metabolic flexibility, adapting to changes in energy intake by modulating energy expenditure. When subjected to a high-fat diet, they enhance this adaptive capacity by increasing complex II activity and maximal mitochondrial respiration in brown adipose tissue, thereby attenuating obesity-related metabolic dysfunction [45].

#### 3.1.3. Leptin and Dyslipidaemia

Adipose tissue is a major source of signalling factors called adipokines, which regulate metabolism. Leptin is an adipokine mainly regulated by oestrogens and insulin. It controls energy balance and metabolism, though its role in cardiovascular regulation remains unclear [46]. Some studies have associated leptins with CVD, particularly HF, whereas others have suggested that leptin may benefit cardiac metabolism [47,48]. Circulating leptin levels are higher in women, likely due to oestrogen regulation and greater body fat. Notably, an association between leptin levels and stroke was observed in women after adjusting for various factors such as age, BMI, and arterial hypertension [32].

Atherogenic dyslipidaemia is characterized by elevated serum TGs; small, dense low-density lipoprotein (LDL); and low levels of high-density lipoprotein (HDL) cholesterol. Premenopausal women typically have a better lipid profile than men, with lower total cholesterol, LDL, and TGs, and higher HDL levels [18]. Nevertheless, after menopause, women experience a shift toward an atherogenic lipid profile similar to that in men, with increased total and LDL cholesterol, TGs, and lipoprotein and decreased HDL levels. This menopause-linked change is proatherogenic and procoagulant, strongly associated with increased VAT and a higher risk of CVD [18].

### 3.2. Insulin Resistance and Dysglycaemia

Insulin, produced by pancreatic beta cells, regulates carbohydrate, lipid, and protein metabolism in adipose tissue, the liver, and skeletal muscle [19]. In obesity, insulin’s effects are impaired, leading to IR, marked by mild hyperglycaemia and hyperinsulinaemia in fasting and postprandial states. IR in adipose tissue disrupts metabolic processes, contributing to ectopic fat accumulation, inflammation, and insulin signalling defects. Men, with greater visceral fat, are more prone to IR than women. In animal models, females are less susceptible to fatty acid-induced IR, and male rodents develop IR more quickly than females on a high-fat diet [19].

Abnormal glucose homeostasis is diagnosed based on the presence of impaired fasting glucose (IFG) (5.6–6.9 mmol/L) or impaired glucose tolerance (IGT) (i.e., 2 h post-challenge glucose concentration of 7.8–11 mmol/L) [49]. IFG relies on hepatic glucose production and insulin sensitivity, while IGT involves insulin secretion in response to carbohydrates. The prevalence of IFG is higher in men, while IGT is more common in women until the age of 60 years in Asia and 80 years in Europe. T2D and glucose abnormalities are also more common in men, who face greater risk of vascular complications. Women with T2D are more likely to experience obesity, hypercholesterolaemia, and hypertension, with BP rising faster with age [16,33]. Although more women have T2D due to a larger elderly population, diabetes in women is linked to a higher risk of cardiovascular death, while men with a history of MI face greater mortality risk than those with diabetes [18].

### 3.3. Non-Alcoholic Fatty Liver Disease

NAFLD is diagnosed when hepatic TG levels (steatosis) exceed 5.5% in the absence or moderate consumption of alcohol, which is defined as less than 20 g per day for women and 30 g for men [50]. Recognised as a multisystemic disease, NAFLD is associated with an increased incidence and prevalence of CHD, independent of age. NAFLD is a common liver disease, and its progression to non-alcoholic steatohepatitis (NASH) considerably increases the risk of severe complications, such as cirrhosis, which can lead to hepatocellular carcinoma [18].

NAFLD could be both a cause and a consequence of MetS. Indeed, NAFLD is closely linked to IR, and its prevalence is five times higher in patients with T2D than in those without T2D [51]. Therefore, NAFLD is often considered to be a hepatic manifestation of MetS [52] and should be renamed “metabolic dysfunction-associated fatty liver disease” (MAFLD), characterized by the presence of hepatic steatosis and either a of either obesity, T2D, or metabolic dysfunction [53].

Men are more prone to NAFLD than women before the age of 50 years. After menopause, women have a similar or even higher prevalence of NAFLD compared to men of the same age. Women with polycystic ovary syndrome (PCOS) or a history of gestational diabetes mellitus (GDM) have a similar or higher risk of developing NAFLD than men [18]. As chronic liver disease progresses, men tend to exhibit more advanced stages of NASH and are more susceptible to fibrosis development than women [34]. However, a higher incidence and prevalence of cardiovascular events and greater cardiovascular and all-cause mortality have been observed in women with NAFLD than in men [35].

### 3.4. Obstructive Sleep Apnoea

OSA represents one of the most significant sleep disorders, with a prevalence of 9–17% among the general population [36]. OSA is characterized by recurrent episodes of apnoea (complete cessation of breathing) and hypopnoea (partial reduction in breathing) during sleep, leading to intermittent hypoxaemia, autonomic fluctuation, and sleep fragmentation [54].

Patients with OSA frequently exhibit conditions associated with MetS, such as arterial hypertension, elevated fasting blood glucose, increased waist circumference, low HDL cholesterol, high TG levels, and IR [55]. This has led to the suggestion that OSA may be a manifestation of MetS [56]. Moreover, intermittent hypoxia in OSA triggers the release of free radicals, leading to oxidative stress, cytokine production, and systemic inflammation [57,58]. These processes may establish a pathogenic link between OSA and key MetS components, including glucose intolerance, IR, hypercholesterolaemia, and hyperlipidaemia [59,60,61,62].

OSA is present in 24% of young men and 9% of young women, increasing to 34% and 17% in middle-aged men and middle-aged women, respectively [36]. Among older adults, 70% of men and 56% of women are affected. The male-to-female ratio of OSA occurrence ranges from 3:1 to 5:1 in the general population [37]. This sexual dimorphism may be explained by the fact that women are less likely to report sleep-related complaints or symptoms and often use different language to describe their sleep-deprived feelings. Young et al. noted that 40% of women with an apnoea/hypopnea index greater than 15 did not report any of the “classic” OSA symptoms, compared to 20% of men. Although women may experience symptoms similar to those of men, they often present with vague and nonspecific symptomatology [38].

### 3.5. Polycystic Ovary Syndrome

PCOS is a complex and heterogeneous endocrine disorder affecting approximately 6–20% of women of reproductive age [63]. This syndrome is characterized by three primary clinical features [64]: (1) hyperandrogenism—either clinical (e.g., hirsutism, acne) or biochemical (elevated androgen levels) or both; (2) oligo-anovulation—irregular or absent ovulation; and (3) polycystic ovarian morphology—identified via ultrasound, defined as the presence of ≥12 follicles (2–9 mm in diameter) or an ovarian volume > 10 mL. PCOS diagnosis requires the presence of at least two of these three criteria. Additionally, other potential causes of hyperandrogenism, such as nonclassical congenital adrenal hyperplasia and hyperprolactinaemia, must be excluded [65]. As PCOS affects women of childbearing age, no sexual dimorphism has been described.

PCOS is not officially classified as part of MetS, but it shares several metabolic dysfunctions associated with this syndrome [63]. Women with PCOS have a prevalence of MetS of approximately 40% [66]. IR has been reported in approximately 50–80% of women with PCOS [67], affecting peripheral tissues such as skeletal muscle and adipose tissue, with abnormalities in insulin receptors [68], compensatory hyperinsulinaemia, dyslipidaemia, and arterial hypertension [64].

Obesity, particularly abdominal obesity, is also prevalent among women with PCOS [69]. When adipose tissue function is impaired, adipocytes secrete abnormal levels of pro-inflammatory cytokines and adipokines, including interleukin (IL)-6, IL-8, tumour necrosis factor alpha (TNFα), leptin, adiponectin, resistin, lipocalin-2, monocyte chemoattractant protein-1 (MCP-1), retinol-binding protein-4, and CXC-chemokine ligand-5, all of which may contribute to IR [70,71].

The familial aggregation of PCOS has been well established, with a range of metabolic abnormalities identified in the parents and siblings of women with PCOS compared with women without PCOS. Indeed, higher prevalences of MetS (in mothers, fathers, and sisters), arterial hypertension (in fathers, sisters, and brothers), and dyslipidaemia (in mothers and fathers) have been observed [72,73]. Sons of women with PCOS also exhibit early metabolic markers, such as an increased waist-to-hip ratio, higher cholesterol levels, and elevated body weight, from infancy to adulthood [74,75].

## 4. Developmental Origins of Cardiometabolic Diseases

Despite the health policies aimed at adulthood over the last few decades, the incidence and prevalence of cardiometabolic disorders are still increasing [76]. This phenomenon can be partly explained by the fact that adverse events in the perinatal period may increase susceptibility to developing these non-communicable diseases in adulthood.

**Figure 5:**
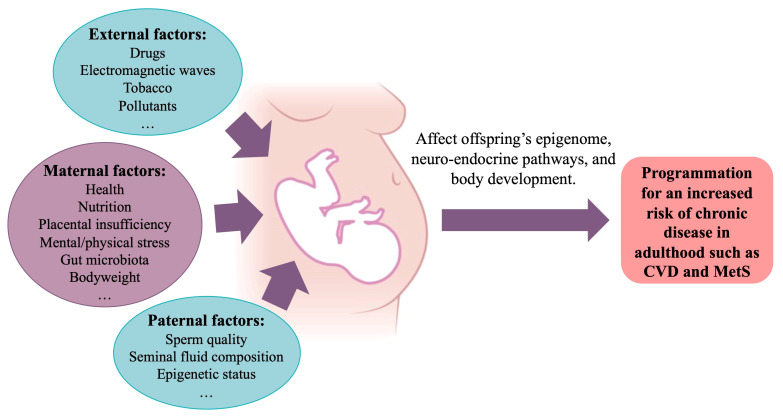
Perinatal stressors that determine greater risk of chronic disease. Maternal and external environmental factors influence the offspring’s susceptibility to developing cardiovascular diseases (CVDs) and metabolic syndrome (MetS) in adulthood, depending on the nature, time window, and duration of exposure time of the insult. Created using a licensed version of BioRender.com.

The concept of DOHaD originated in the 1980s with the publication of an epidemiological report by Professor Barker’s, who observed an inverse correlation between the standardized mortality of ischaemic heart disease and individual birth weight [77]. The DOHaD concept proposes that a change in the environment during a critical developmental period early in life can induce permanent changes in the structure and function of certain organs, potentially leading to the later development of chronic diseases [78]. This critical period of vulnerability is currently considered to include the first thousand days of life, from the periconceptional period until the second birthday [79]. During this time window, exposure to stress factors such as altered maternal nutritional status, toxic substances, or gestational complications that perturb the in-utero environment may predispose the individual to the later onset of cardiometabolic disease (Figure 5). In addition, recent data have shown that paternal conditions, such as sperm quality, epigenetic status, and seminal fluid composition, may also influence the development of these diseases [80].

Low-birth-weight individuals, as a consequence of preterm birth or IUGR, are particularly at risk of developing these chronic diseases later in life. However, it is well established that the risk of developing these pathologies has a parabolic pattern, indicating that individuals born with a high birth weight are also at risk [81].

### 4.1. Preterm Birth

**Figure 6:**
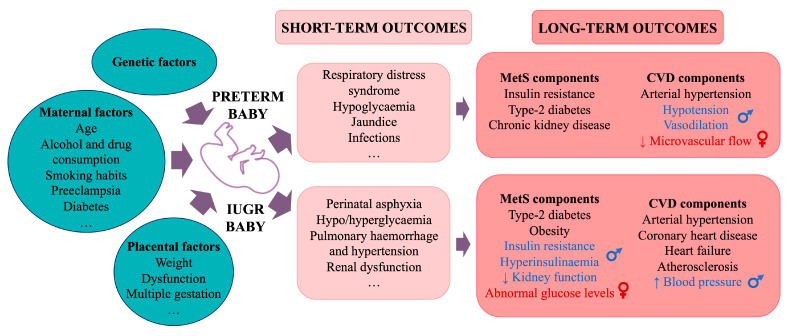
Risk factors of preterm birth and intrauterine growth restriction (IUGR) development with short- and long-term outcomes. Preterm birth and IUGR results for many factors and their combinations. Afflicted neonates can encounter complications after birth, which are considered “short-term outcomes”, as well as various adult diseases, including metabolic syndrome (MetS) and cardiovascular disease (CVD) components, which are considered “long-term outcomes”. Some of these diseases present sexual dimorphism in their importance and prevalence. Created using a licensed version of BioRender.com.

Preterm infants, born alive before 37 weeks of gestation, accounted for 13.4 million births in 2020 [82]. Several maternal factors are linked to preterm birth, including conditions like GDM, placental issues, preeclampsia, infections, multiple gestations, and substance use [83]. Preterm births are associated with various short-term health issues, such as respiratory distress, infections, and jaundice, among many others [84]. Additionally, adults born preterm have higher rates of MetS and CVD components, including IR, T2D, arterial hypertension, and chronic kidney disease, later in life [85,86,87,88,89] compared to those born full-term (Figure 6).

### 4.2. Intrauterine Growth Restriction

Intrauterine growth restriction (IUGR), affecting 10–15% of births, occurs when a foetus fails to reach its genetic growth potential [90]. It can result from maternal, placental, foetal, or genetic factors, often acting in combination [91]. IUGR can lead to complications at birth, including perinatal asphyxia, feeding issues, and pulmonary or renal problems [91]. Long-term, IUGR increases the risk of developing cardiovascular and MetS components such as obesity and T2D. IUGR is also linked to arterial stiffness and to higher risk of atherosclerosis, CHD, HF, and arterial hypertension development in adulthood [90,92,93] (Figure 6).

### 4.3. Sexual Dimorphism in Cardiometabolic Disorders Related to Low-Birth-Weight Individuals

The risk of preterm birth, very preterm birth, and extremely preterm birth is greater for male foetuses than for females [94]. In 1989, Barker et al. reported that, among men, those with the lowest body weights at birth and at 1 year of age had the highest death rates from ischaemic heart disease, providing a first link between early growth patterns of individuals and their susceptibility to chronic disease in later life [95]. In 1993, Osmond et al. published the first study reporting a positive association between low birth weight and the risk of CVD in men and women, which was stronger in men than in women [77].

Hypotension and low cardiac output are frequently observed during the first 48 h of life in very premature infants (29 weeks of gestation) [96], particularly in premature boys compared to girls [97], which could be due to alterations in the regulation of vascular resistance and impaired microvascular vasodilation [98]. Very preterm boy babies have more vasodilation than girl babies of the same gestational age at 24 h of life [99]. Sex-specific differences have also been observed in peripheral microvascular blood flow in preterm infants [100].

The control of neonatal vascular tone also shows sexual dimorphism, such that boy babies are at a greater risk of developing several complications following premature birth [101]. Girls exhibit higher levels of norepinephrine, a key sympathetic neurotransmitter responsible for regulating the cardiovascular system and linked to lower microvascular flow [102]. In contrast, male preterm infants have higher levels of vasodilators such as nitric oxide (NO) and carbon monoxide (CO), which may contribute to excessive vasodilation [100].

Small size (weight and length) at birth is associated with increased IR and hyperinsulinaemia in young adulthood, predominantly in men [103]. Research from Australia found that women with a low body weight have a greater incidence of abnormal blood glucose levels compared to men, increasing their risk of developing T2D [104]. Additionally, IUGR is associated with elevated BP and reduced kidney function in early adulthood (20- to 30-year-olds), with a more significant impact in men [105].

### 4.4. Macrosomia and Large for Gestational Age

**Figure 7:**
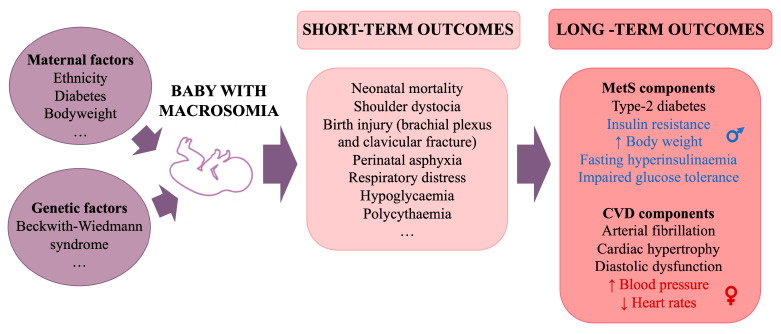
Risk factors of macrosomia development with short- and long-term outcomes. Babies born with macrosomia are exposed to a variety of direct complications considered “short-term outcomes”. Various other diseases, including metabolic syndrome (MetS) and cardiovascular disease (CVD) components, may appear later in life, considered “long-term outcomes”. Some of these diseases present sexual dimorphism in their importance and prevalence. Created using a licensed version of BioRender.com.

The proportion of infants who are large for gestational age (LGA) is increasing [106], in conjunction with the global increase in obesity. LGA is defined as a birth weight > 90th percentile for gestational age (GA), and macrosomia is defined as a birth weight > 4000 g or 4500 g, regardless of GA [107]. Factors contributing to excessive foetal growth include genetic conditions, such as Beckwith–Wiedemann syndrome and ethnicity (with macrosomia more common among White newborns compared to Black or Hispanic newborns), and maternal factors, such as T2D, pre-pregnancy weight, and excessive maternal weight gain [108]. Short-term complications include a greater risk of neonatal mortality, shoulder dystocia, birth injuries such as brachial plexus injury and clavicular fracture, perinatal asphyxia, respiratory distress, hypoglycaemia, and polycythaemia [109]. Macrosomia also has long-term metabolic effects. In adulthood, individuals with a history of macrosomia are at greater risk of obesity, T2D [110], IR, and CVD; notably increased risk of arterial fibrillation, cardiac hypertrophy, and diastolic dysfunction [111]; and greater risk of increased radial and carotid artery intimal thickness [112] (Figure 7).

### 4.5. Sexual Dimorphism in Cardiometabolic Disorders Related to Individuals with Macrosomia

Sexual dimorphism is present in the development of cardiometabolic disorders in individuals with a history of macrosomia. Adult males with neonatal macrosomia tend to have significantly increased body weight, reduced voluntary activity, IR, fasting hyperinsulinaemia, and IGT. In contrast, adult females with neonatal macrosomia do not typically show significant changes in body weight or endocrine profiles relative to other females, although they may have higher BP and lower heart rates [113].

## 5. Mechanisms Potentially Involved in Sexual Dimorphism in Cardiometabolic Diseases

**Figure 8:**
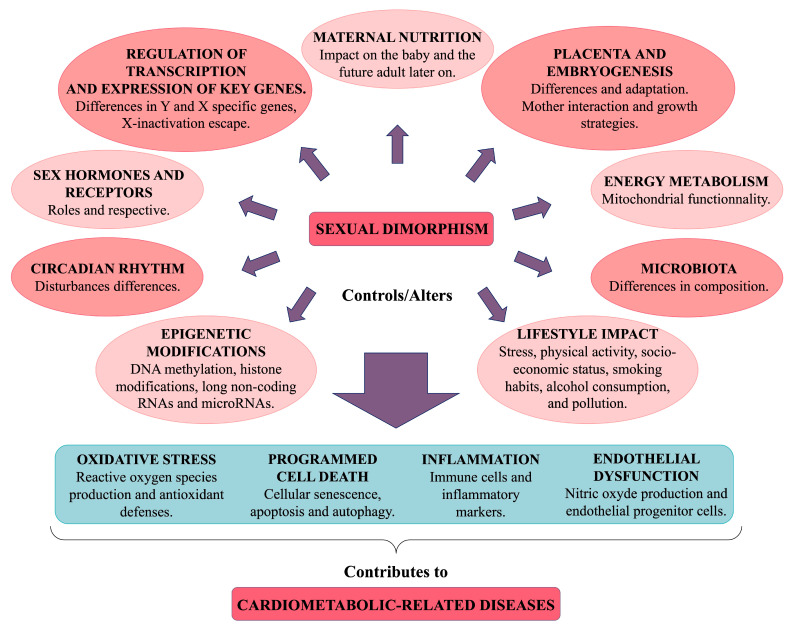
Mechanisms potentially involved in the sexual dimorphism of cardiometabolic diseases. Genetic differences, sex hormones, lifestyle choices, early development, and metabolism are important factors shaping sexual dimorphism. Different cellular stress responses and reactions resulting from these factors can contribute to cardiometabolism-related diseases.

Sexual dimorphism in health and disease arises from genetic and hormonal differences, as well as interactions with environmental factors including diet, medication, disease, and stress. An individual’s phenotype results from complex interactions between genotype and both current and past environmental influences, leading to lifelong epigenomic remodelling. Many common diseases exhibit sex-specific differences that are often rooted in early development, particularly cardiometabolic disorders. This sexual bias can be attributed to the role of sex chromosomes, the regulatory pathways involved in sexual development, fluctuations in sex hormones, circadian rhythms, maternal nutrition, energy metabolism, and epigenetic and lifestyle influences [103]. The cumulative effects of these regulatory factors may influence endothelial dysfunction, cell death/senescence, inflammation, and oxidative stress (Figure 8). Consequently, the same condition may develop through different pathways in men and women, implying that therapeutic targets may also differ [8].

### 5.1. Genetic Factors

It is well established that certain individuals are genetically more susceptible to CVD and its associated risk factors. Heritability is a significant factor in arterial hypertension, with genetics accounting for 30% to 50% of hypertension risk [114]. Genome-wide association studies have identified numerous genetic variants associated with BP regulation [115]. A family history of CVD further amplifies the risk of developing the condition [116]. Sex chromosome composition may explain the sexual biases in congenital heart defects, influencing their presentation and associated mortality and morbidity [8]. The expression of Y and X chromosome-specific genes, along with X-chromosomal gene escape from X inactivation, contributes to sex differences in the transcriptome [117]. Hundreds of genes exhibit sex-specific expression patterns in a tissue-specific manner [118]. In patients with aortic stenosis, significant sex differences were observed in genes for collagen I (*COL1A1*), collagen III (*COL3A1*), and matrix metalloproteinase 2 (*MMP2*), which are involved in fibrosis and inflammation regulation, with repression of these processes in women. Men typically exhibit higher levels of the *carbonic anhydrase 3* gene, which is associated with hypertrophy and HF, and decreased expression of the *APOJ/clusterin* gene, which is believed to regulate autophagy and protect against inflammatory damage [119]. In HF and other diseased conditions, females have higher expression of genes related to energy metabolism than males do [3], suggesting that females may be able to maintain their metabolic function more effectively in response to illness. Additionally, some allele mutations linked to CVD risk are sex-specific. For example, about 70 distinct gene loci contribute to the adipose phenotype, with some polymorphisms acting in a sex-specific manner [120,121].

#### 5.1.1. Y and X Chromosome-Specific Genes

Approximately 30% of trophoblast-expressed genes are located on the X chromosome. Inactivation of paternal X in trophoblast cells can lead to embryonic death when a mutant allele is maternally transmitted in mice [122]. Studies have shown that female mammalian embryos, which possess two X chromosomes, survive better under heat and oxidative stress, correlating with increased expression of X-linked housekeeping genes. These genes include *glucose-6-phosphate dehydrogenase*, which is essential for the production of reducing equivalents that resist oxidative stress, and inhibitors of caspase activity (e.g., *XIAP*) [119]. Furthermore, an X-chromosome-linked gene with two copies in females and one in males has been implicated in protecting against cardiac myocyte apoptosis [123,124].

X-linked genes are also implicated in the sexual dimorphism observed in inflammatory biomarker expression, as the human X chromosome contains many immune response-related genes, including *IL-2 receptor-γ chain*, *IL-3 receptor-α chain*, *IL-9 receptor*, *IL-13 receptor-α chains*, *Toll-like receptor 7 and 8*, and *IL-1 receptor-associated kinase 1*, as well as multiple transcriptional and translational effectors [125]. This suggests that the X chromosome’s contribution to sex differences in cardiovascular phenotypes may have been underestimated, especially since it has often been excluded from genome-wide association studies [3].

About 95% of the Y chromosome is a male-specific region and does not recombine with the X chromosome during meiosis, meaning that it is directly inherited from father to son. Several linked genes have been associated with cardiovascular risk factors such as arterial BP and LDL cholesterol [13]. In addition, variants of Y chromosome loci have been found to affect cardiac function, and some Y-linked genes (*TBL1Y* and *KDM5D*) have been implicated in cardiac differentiation in human embryonic stem cells [3,8]. Similarly, the Y-chromosome-specific *UTY/Ddx3y* gene has been implicated in significant genetic variation in the cardiac left ventricle in mice [123].

#### 5.1.2. Dosage and Compensation of Gene Expression and X-Inactivation Escape

Dosage and compensation mechanisms between males (XY) and females (XX) are tightly regulated but unequal. Females must avoid X-linked gene overexpression by inactivating one of their two X chromosomes, a process known as X inactivation. Simultaneously, both sexes require balanced expression between X-linked and autosomal genes, which is achieved through the transcriptional upregulation of the active X chromosome [103].

X-inactivation escape, in which some X-linked genes avoid inactivation, can occur as early as the 4–8-cell stage of embryonic development or during aging [126]. It has been estimated that between 15% and 24% of the 1400 human X-linked genes escape X-chromosome inactivation, resulting in a higher copy number in women [122,125]. The degree of X-inactivation escape can vary significantly, ranging from 5% to 75%, depending on the specific loci; it also varies across different tissues and individuals [122]. For example, sexual dimorphism in gene expression is present in 14% of expressed genes in the brain and up to 70% in the liver [103].

Research suggests that the presence of two X chromosomes in cardiovascular cells might increase female susceptibility to ischaemia/reperfusion injury [8]. Moreover, female polymorphisms in X-linked genes may not be expressed uniformly across all tissues as they are in males, leading to greater variability in female physiological responses. Unfortunately, the variability of female physiological responses is often used to justify the exclusion of female animals from many basic science studies, even though this variability can be accommodated with appropriate experimental design [13].

### 5.2. Epigenetic Regulation

**Figure 9:**
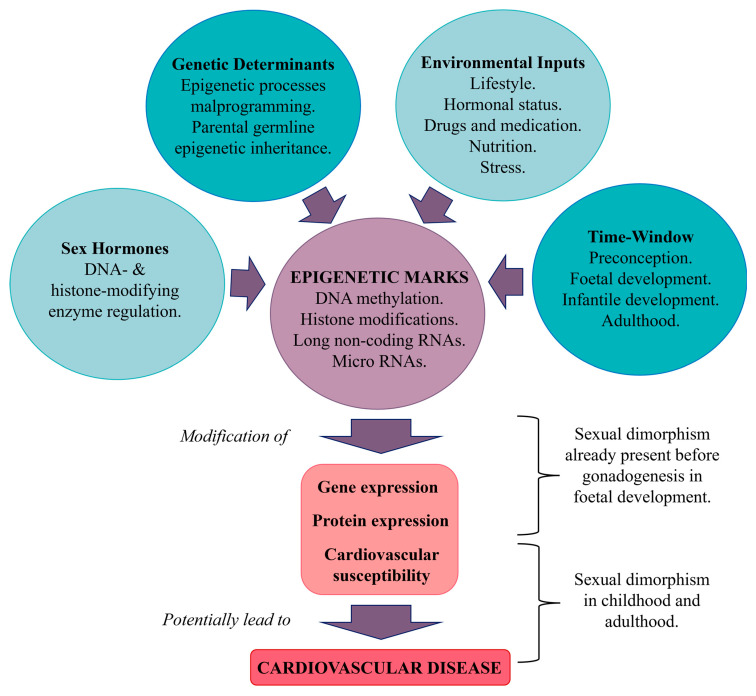
Epigenetic influence on the sexual dimorphism of cardiovascular susceptibilities. Genetic determinants, environmental inputs, and sex hormones, as well as the time-window shape, influence epigenetic marks, potentially leading to transgenerational effects. Therefore, epigenetic marks impact sexual dimorphism before gonadogenesis but also throughout the lifetime, which results in differential cardiovascular susceptibility, potentially leading to cardiovascular disease.

Epigenetic marks, which reflect lifestyle, nutrition, stress, hormonal status, drugs, and medications, can influence gene expression in a sex- and gender-specific manner [14] (Figure 9). Notably, human pluripotent stem cells exhibit different sex-related developmental trajectories when differentiating into cardiac progenitors. This sex-specific differentiation involves both transcriptional and epigenetic biases that are established shortly after fertilization and persist in cardiac lineages. These findings suggest that early epigenetic events continue beyond embryogenesis, indicating that the cardiac genome is functionally distinct between sexes, even before gonadogenesis. As a result, male and female cardiac precursor transcriptomes are not equivalent in terms of gene expression and epigenetic differences. Network analyses have revealed significant differences in regulatory structures between the sexes, with certain transcription and epigenetic factors being differentially expressed in adult hearts [8]. This raises questions about how the dosage of these factors might affect their downstream targets.

Interestingly, many transcription factors, such as oestrogen (ERα and ERβ) and androgen receptors, have sex-biased gene targets in various tissues, despite not being differentially expressed themselves. This fact may be due to epigenomic sex differences, which may influence the accessibility of transcription factors to their specific motifs. Therefore, the same transcription factor may regulate different sets of genes in male and female cells, leading to distinct gene expression profiles in the two sexes [8].

#### 5.2.1. Epigenetic Marks and Their Influence

Epigenetic marks, due to their inherent flexibility, enable environmental factors to alter the entire genome or specific gene regions [103]. These marks primarily include DNA methylation (of cytosines), histone modifications (such as acetylation and methylation), and non-coding RNAs [117]. During critical periods of life (specifically, preconception and foetal and infantile development), environmental influences such as nutrition, drugs, toxins, endocrine disruptors, and cultural factors can impact these epigenetic marks in a sex-specific manner [103] and can have lasting consequences, affecting the development and function of various tissues and ultimately influencing the phenotype and susceptibility to disease [122].

#### 5.2.2. DNA Methylation

DNA methylation has been linked to cardiometabolic diseases. A systematic review of 25 studies identified 31 gene methylation sites associated with sex differences [127]. These genes were primarily related to plasma lipoproteins, cholesterol, and statin response [127]. Sex hormones can regulate DNA- and histone-modifying enzymes that have an influence on genes related to CVD. To illustrate, DNA-modifying enzymes, such as histone acetyltransferase CREB-binding protein, are known to be recruited to the DNA by oestrogen and androgen receptors [117]. These receptors act by binding to hormone response elements in the DNA and attract various cofactors that have inherent histone acetyltransferase or methyltransferase activity. The histone-modifying enzymes alter the epigenetic state of gene promoters, thereby changing gene expression.

Epigenetic modifications have been strongly implicated in the regulation of CVD-related genes. Specifically, the balance of histone acetylation, controlled by histone acetyltransferases and deacetylases, is crucial for the management of cardiac hypertrophy. Dysregulation of histone methylation profiles has been observed in HF, indicating that these modifications are critical in the pathology of CVD [3]. Two notable genes, *kdm5c* and *kdm6a*, which are X-escapee genes that demethylate H3K4me3 and H3K27me3, respectively, are known to be expressed in a sexually dimorphic manner in cardiac tissue. As they are involved in the epigenetic modification of inflammatory mediators such as microglia, it has been hypothesized that they play critical roles in the mediation of stroke sensitivity [126].

#### 5.2.3. Long Non-Coding RNA

Long non-coding RNA (lncRNA) refers to RNA transcripts more than 200 nucleotides in length. These RNAs do not contain a protein-coding sequence and display greater tissue-specific expression and function [128]. They play a major role in cardiovascular and metabolic differentiation, development, homeostasis, and pathophysiology and are considered biomarkers for CVD. Thus, lncRNA may be involved in the morphological, physiological, and functional differences observed between men and women and may, therefore, be differentially expressed in the two sexes [129].

In humans, the most important contribution of lncRNA to sex differences is provided by the lncRNA X-inactive specific transcript (*XIST*), encoded on the X chromosome, which contributes to the coating and the random inactivation of one of the two X chromosomes in females [130]. In the cardiovascular system, RNA-seq analysis identified a distinct expression profile between sexes of messenger RNA (mRNA) and lncRNA in both atria and ventricles [131]. In particular, one of the genes included in the lncRNA *KCNQ1OT1* imprinted cluster, cyclin-dependent kinase inhibitor 1C (*CDKN1C*), has been identified as a novel female-specific biomarker of left ventricular dysfunction after MI [132].

Concerning metabolism regulation, lncRNA *H19* regulates glucose homeostasis, β-cell function, and body weight [133,134]. Low levels of *H19* have been associated with IR and T2D [135]. In skeletal muscle in particular, greater expression of *H19* in females was associated with improved insulin sensitivity [136] and, thus, higher glucose uptake [137]. However, higher expression of *H19* has been found to be related to the shorter lifespan observed in male subjects [138].

#### 5.2.4. MicroRNAs

MicroRNAs (miRNAs) are non-coding RNA molecules that regulate gene expression by targeting mRNAs, leading to their degradation or the repression of translation by interacting with the 3′ untranslated region (UTR). Interaction with other regions, including the 5′ UTR, coding sequence, and gene promoters, is also possible. In addition to their intracellular functions, miRNAs also serve as extracellular messengers, facilitating intercellular communication [139].

Sex-specific differences in miRNA profiles have been observed in various cardiovascular conditions. For example, in patients with coronary artery calcification, there are distinct differences in miRNA expression in men and women, suggesting that oestrogen-dependent regulation of miRNAs may contribute, particularly in cardiac fibrosis [140]. Studies have shown that miRNAs, such as miR-21, miR-24, miR-27a/b, and miR-106a/b, are upregulated in males and regulated by oestrogens and ERβ. These miRNAs, which are located on three repressors (*Rasa1*, *Rasa2*, and *Spry1*) of the mitogen-activated protein kinase (MAPK) signalling pathway, promote processes that can lead to fibrosis [3]. Oestrogens also influence other miRNA clusters, such as miR-17-92, and affect the expression of miR-221/222, which can modulate ER levels [141]. Notably, elevated levels of circulating miR-221 and let-7g have been detected in women with MetS, potentially increasing the risk of developing CVD [142]. These sex-dependent differences in miRNA expression are also apparent in normal and diseased cardiac tissues across species. Aged female rats have increased expression of miR-15a, miR-19b, miR-32, miR-136, and miR-199a-3p following stroke compared to males [143]. In mice, a sex-dependent difference in miRNA expression was observed in the normal heart and in ischaemic cardiomyopathy: indeed, 13 mouse miRNAs are sexually dimorphic in ischaemic cardiomyopathy and 6 in the normal heart. In humans, 3 and 15 miRNAs were found to be sexually dimorphic in ischaemic cardiomyopathy and in the normal heart, respectively [144].

### 5.3. Maternal Nutrition

Maternal nutrition has an essential role in the growth of offspring during critical periods of development, such as pregnancy and lactation, influencing gene expression, the epigenome, metabolism, and cellular function [145].

#### 5.3.1. Maternal Overnutrition

In humans and animals, maternal overnutrition during pregnancy and/or lactation predisposes offspring to develop obesity and increases the risk of MetS, IR, and arterial hypertension [146,147]. A high maternal BMI before and during pregnancy is a predictor of offspring obesity, adiposity, and MetS thereafter [148]. Studies utilizing HFDs in animal models have also provided insight into the effects of maternal overnutrition during the prenatal period. Mothers given an HFD produce milk with significantly higher energy content than those receiving low-fat diets, leading to heavier offspring [149] with elevated leptin levels and IGT [150]. Additionally, increased sugar consumption has been associated with rises in obesity rates and related metabolic complications [151,152]. Moreover, a study published by Gracner et al. indicated that early-life sugar restriction, during the first 1000 days of life, can reduce the risks of T2D and hypertension by approximately 35% and 20%, respectively, delaying onset by 4 and 2 years, respectively, with the most significant benefits seen when rationing extended beyond six months after birth [153].

**Figure 10:**
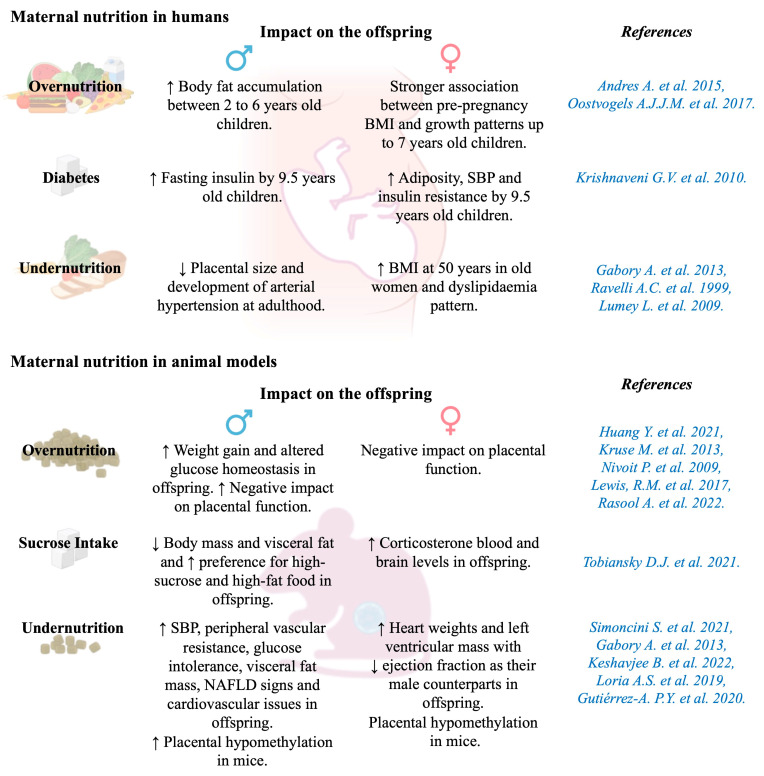
Impact of maternal nutrition on placenta and offspring according to sex. Figure showing the different long-term impacts of maternal overnutrition, diabetes, and undernutrition on offspring according to sex [25,103,154,155,156,157,158,159,160,161,162,163,164,165,166,167]. Abbreviations: BMI, body mass index; MetS, metabolic syndrome; NAFLD, non-alcoholic fatty liver disease; SBP, systolic blood pressure. Created using a licensed version of BioRender.com.

Sexual dysmorphism related to the effects of maternal overnutrition has been observed in several studies (Figure 10). For example, male children born to obese mothers during pregnancy tend to accumulate more body fat between the ages of 2 and 6 years than those born to normal-weight or overweight mothers [154]. A stronger association was observed between maternal pre-pregnancy BMI and growth patterns up to 7 years of age in girls than in boys [155]. Additionally, in a study conducted in India, female children of diabetic mothers were found to exhibit higher adiposity, SBP, and IR than female children of non-diabetic mothers at the age of 9.5 years, while male children of diabetic mothers had higher fasting insulin than male children of non-diabetic mothers at the same age [156].

Animal studies that have explored both sexes have identified that male offspring generally exhibit greater sensitivity to metabolic disturbances induced by maternal overnutrition, such as weight gain and altered glucose homeostasis, than their female counterparts [147,157]. Maternal HFD intake, in particular, has been linked to greater weight gain and altered glucose regulation in male offspring [158], as well as an increased predisposition to liver steatosis and related inflammatory markers [168]. Additionally, maternal sucrose intake has differential effects on offspring; in males, it is associated with reduced body mass and visceral fat while increasing dietary preferences for high-sucrose and high-fat food, and in females, it is associated with raised blood and brain corticosterone levels in adulthood [159].

#### 5.3.2. Maternal Undernutrition

The long-term consequences of maternal undernutrition were well illustrated by the Dutch famine birth cohort (1943–1947), which included 2414 singletons born alive and at term in the Wilhelmina Gasthuis hospital in Amsterdam during the Dutch famine [160]. During this period, exposure to famine during gestation was defined as an average maternal daily ration of fewer than 1000 calories. Three 16-week periods were identified: children mainly exposed to maternal undernutrition during late gestation, mid gestation, or early gestation. The BMI of 50-year-old women whose mothers were exposed to famine in early gestation was 7.4% higher than that of children of unexposed mothers but did not differ significantly in women whose mothers were exposed during the mid- or in late-gestation periods. In 50-year-old men, prenatal exposure to famine did not significantly impact BMI [161]. However, women of about 58 years old who were exposed to famine in utero exhibited a dyslipidaemia pattern, with elevated levels of cholesterol (0.26 mmol/L; 95% CI: 0.07, 0.46; *p* = 0.007), triglycerides (0.17 mmol/L; 95% CI: 0.03, 0.31; *p* = 0.02), and LDL cholesterol (0.17 mmol/L; 95% CI: –0.01, 0.36; *p* = 0.06) compared to those who were not exposed. This dyslipidaemia pattern was not observed in men [162].

In newborn human and animal models, intrauterine calorie restriction is associated with growth retardation and long-term outcomes that are sexually dimorphic (Figure 10). Prospective longitudinal studies in humans have shown that a lower protein-to-carbohydrate ratio in the maternal diet is associated with higher SBP in 4-year-old children of both sexes [163]. In animal models, low-protein diets fed to mothers throughout gestation result in IUGR in the offspring. Male offspring have higher SBP and peripheral vascular resistance, glucose intolerance, increased visceral fat mass, and some signs of NAFLD [25,164]. They also exhibit other cardiovascular issues, such as neonatal cardiac dysfunction, pathological vascular remodelling, and increased cardiovascular sympathetic tone and contractile responses [163]. Finally, in 22-month-old rats born in a maternally undernourished environment, males and females both had larger heart weights and left ventricular mass, with a lower ejection fraction, than control rats, but only males had elevated BP values [165]. In baboon foetuses, moderate maternal undernutrition (70% of the diet) led to IUGR. IUGR males, but not females, displayed left ventricular fibrosis and concentrations of pro-inflammatory factor Nuclear Factor Kappa B Subunit 1 that were inversely correlated with birth weight [169]. Impaired supply of micronutrients (notably, perinatal iron deficiency) was shown to alter the modulation of vascular tone and, therefore, increased the risk of developing CVD only in male offspring [170].

### 5.4. The Placenta and Embryogenesis

#### 5.4.1. The Placenta

**Figure 11:**
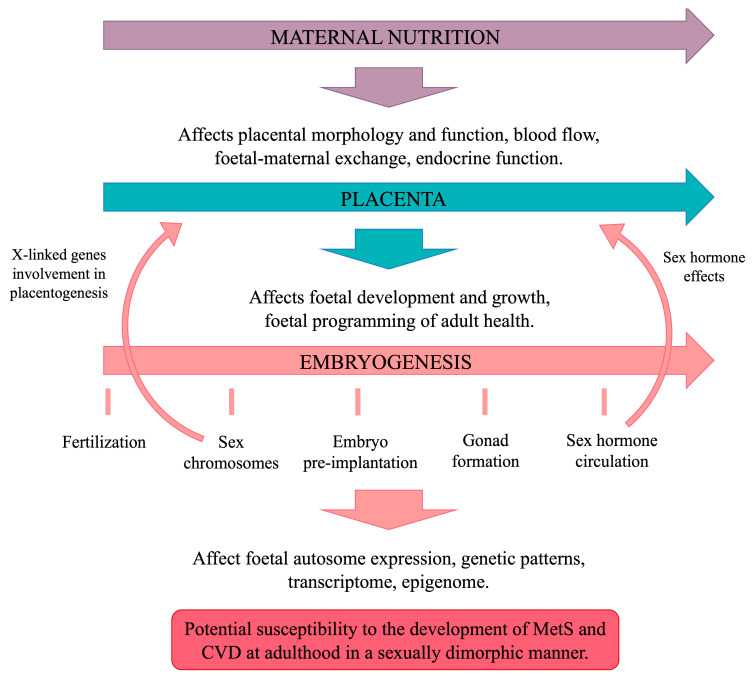
Interconnection between maternal nutrition, the placenta, embryogenesis, and cardiometabolic risk. Maternal nutrition influences elements such as blood flow and nutrient exchanges with the placenta. Depending on those parameters, the status of the placenta affects foetal development and growth, with potentially long-lasting consequences. Finally, sex chromosomes and embryogenesis-related hormones can influence the placenta and create a feedback loop. The potential consequences of poor maternal nutrition can lead to susceptibility to stressors and the development of metabolic syndrome (MetS) and cardiovascular diseases (CVDs) in adulthood.

The placenta plays a crucial role in mediating maternal–foetal exchange, facilitating gas, nutrient, and waste transport while synthesizing hormones. Maternal conditions can impair placental function, affecting the structure, blood flow, and endocrine activity of the foetus, which, in turn, influence foetal growth (Figure 11). These effects vary based on developmental stage, stressor type, and foetal sex [122]. Women carrying male foetuses are more prone to chronic placental inflammation, preterm birth [171], and placental abruption, while those carrying females face a higher risk of placental accreta [172].

Since the placenta shares the foetus’s genetic sex, it exhibits sex-specific sensitivity to hormones, influencing gene expression. X-linked genes significantly impact placental function, particularly in amino acid transport and metabolism, even before adrenal and gonadal development. These sex-specific differences shape the epigenome, affecting long-term gene expression and disease susceptibility. Male placentas are more hypermethylated, suggesting differing strategies to optimize placental health between sexes [122].

Maternal obesity early in pregnancy harms placental function through lipid accumulation, mitochondrial dysfunction, and oxidative stress [166], with more severe effects in male foetuses [167]. Studies on Dutch famine survivors found that maternal undernutrition reduced placental size, disproportionately affecting boys, and was linked to adult arterial hypertension in men but not women [122] (Figure 10).

Sexual dimorphism in placental function is evident in animal studies. Male placentas show increased maternal blood sinusoids and trophoblast volume, enhancing blood flow, while female placentas exhibit a higher labyrinth-to-junctional zone ratio, optimizing nutrient extraction [173]. Caloric restriction (CR) in pregnancy impairs placental function and induces IUGR, with stronger effects in males. Gabory et al. [122] found calorie-restricted placentas were hypomethylated (more so in males), affecting lipid metabolism and nervous system genes in females and cell morphology in males. Imprinted and microRNA-coding genes also showed sex-specific methylation responses to CR [122].

#### 5.4.2. Embryogenesis

Sex chromosome-linked genes are expressed shortly after fertilization, influencing autosomal expression and epigenetic patterns. Notably, sex differences emerge in preimplantation embryos and embryonic stem cells of mice and humans [8]. Male embryos exhibit faster growth but slower organ development, which may reduce their adaptability to adverse uterine conditions. This difference in developmental pace could explain why various maternal factors are linked to a greater increase in CVD risk in males than in females, suggesting that female embryos might possess greater resilience in cardiogenesis, potentially due to the demands of later pregnancy [8]. Female foetuses generally show modest growth changes and better adaptation to adverse conditions such as undernutrition or maternal asthma. In contrast, male foetuses often display minimal changes in gene expression in response to these stressors, leading to poorer adaptation and divergent growth patterns [125]. This disparity has led to the hypothesis that male embryos may be more vulnerable to developmental disorders due to less optimal foetal–maternal interactions [8].

### 5.5. Sex Hormones

Sex hormones, synthesized early in embryonic development, are produced primarily in the gonads but also in other tissues. These hormones, part of a large family of endogenous signalling molecules, modulate cellular processes by regulating gene expression and modifying proteins.

Oestrogens, crucial steroid hormones, bind to two nuclear receptors, ERα and ERβ, as well as to G protein-coupled receptor 1 (*GPER1*). ERα is mainly found in the ovaries, uterus, pituitary gland, liver, hypothalamus, bone, mammary glands, cervix, lungs, and vagina. ERβ is mainly expressed in the ovaries, testes, adrenal glands, lymph nodes, spleen, fat tissue, heart, brain, bone, lungs, prostate, gut, and bladder [174].

Testosterone, the most common androgen, binds to androgen receptors located on the X chromosome. This binding induces conformational changes, enabling the receptors to bind to androgen response elements and regulate gene expression [175]. Androgen receptors are expressed not only in reproductive organs, such as the prostate, ovaries, and testes, but also in the liver, endometrium, fat, eyes, bladder, gut, stomach, muscle, lungs, and kidneys. Moreover, testosterone can be converted into oestrogens in both males and females via the aromatase enzyme, which is present in various extragonadal tissues, such as adipose tissue, the brain, bone, heart, and blood vessels [3].

#### 5.5.1. Effects of Oestrogens in Females

**Figure 12:**
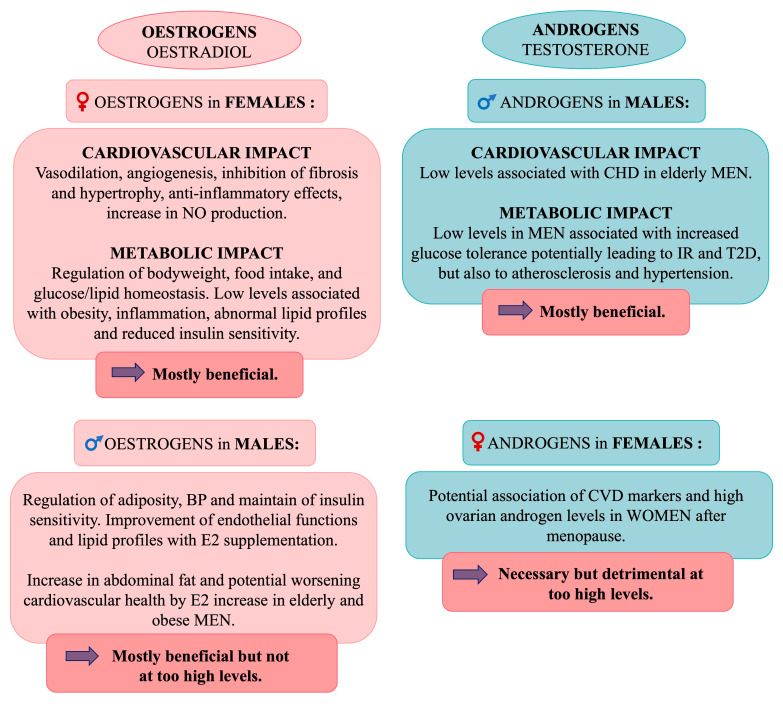
Involvement of sex hormones in cardiometabolic health and disease according to sex. Oestrogens and androgens play different roles in males and females. Oestrogens are mostly cardioprotective in females but can have detrimental effects in males at high concentrations. Androgen effects are beneficial in males at physiological concentrations but have detrimental effects in females after menopause. Abbreviations: BP, blood pressure; CHD, coronary heart disease; CVD, cardiovascular disease; E2, oestradiol; IR, insulin resistance; NO, nitric oxide; T2D, type 2 diabetes.

Oestrogens exert regulatory effects through genomic and non-genomic pathways, involving oestrogen receptor-dependent or -independent mechanisms. These effects in women trigger specific signalling cascades, including those related to Akt, MAPK, NO synthase (NOS), and other enzymes [176]. Non-genomic actions are rapid, causing changes within minutes via the activation of ERs and GPER1, influencing pathways such as ERK1/2, JNK, PI3K, and mTOR [3,14]. Oestrogens, especially oestradiol (E2), which is the major oestrogen, play a cardioprotective role by promoting vasodilation and angiogenesis and inhibiting fibrosis and hypertrophy; they also have metabolic and anti-inflammatory benefits [176]. In addition, oestrogens stimulate angiogenesis by binding to ERα and Erβ on endothelial cells, enhancing VEGF transcription, and increasing NO production via PI3K and Akt activation [117]. Furthermore, oestrogens are involved in the regulation of metabolism, with low oestrogen levels linked to obesity, inflammation, abnormal lipid profiles, and reduced insulin sensitivity [177,178] (Figure 12).

#### 5.5.2. Effects of Oestrogens in Males

The role of oestrogens in cardiovascular health extends beyond females. In males, oestrogens are produced in extragonadal sites, where aromatase converts them to oestradiol without altering circulating levels [173]. In animal models, male mice lacking aromatase have increased adiposity and elevated blood pressure. Similarly, men with aromatase deficiency have lower levels of HDL cholesterol and higher levels of LDL cholesterol and TG [179]. In healthy men, E2 levels influence Apolipoprotein E (ApoE), SBP, and diastolic blood pressure [180]. E2 also helps maintain insulin sensitivity with testosterone [181]. Oestrogens impact adipose tissue distribution, and levels increase in obese men because of the presence of aromatase in fat. In elderly men, E2 levels are higher than in women, possibly worsening cardiovascular health, although oestrogen supplementation improves endothelial function and lipid profiles in men over 65 years of age [182,183]. Age-related changes in Erα/β ratios due to altered oestrogen levels may contribute to arterial hypertension and vascular damage by increasing oxidative stress and inflammation [184]. Men exhibit greater activation of inflammatory and fibrotic markers in left ventricle remodelling under pressure, likely due to sex-specific regulation of collagen I and III mRNA by ERα and ERβ. In female rats, ERα downregulates these collagen levels, while they are upregulated by ERβ in male rats [117] (Figure 12).

#### 5.5.3. Effects of Androgens in Males

Testosterone is associated with several physiological advantages in males, including larger body size, stronger bones, greater muscle mass, and a larger heart, compared to females. Testosterone is closely linked to cardiometabolic benefits in men, as shown by the observation that gonadectomized male mice develop a metabolic profile similar to that of females [185]. In older men [186], low androgen levels are associated with an increased risk of CHD [183]. Additionally, men with lower testosterone levels tend to have increased glucose tolerance [187], potentially contributing to IR and the subsequent development of T2D [188]. Altered androgen levels have also been linked with atherosclerosis and hypertension in men [183,189]. Furthermore, the accumulation of adipose tissue in men enhances aromatase activity, which converts testosterone to E2, leading to further decreases in testosterone levels and increased abdominal fat deposition [190] (Figure 12).

#### 5.5.4. Effects of Androgens in Females

Given that nearly all organs, including cardiovascular tissues, express androgen receptors and respond to androgens, it has been proposed that the increased cardiovascular mortality observed in women after menopause might be linked to elevated ovarian production of testosterone. This increase in testosterone is thought to be stimulated by high levels of circulating gonadotropins during menopause [3]. Indeed, studies have found that higher androgen levels in early menopause are associated with markers of subclinical atherosclerosis [191] and increased carotid intima-media thickness [192]. Nevertheless, further research is necessary to confirm this hypothesis. Additionally, androgen receptor gene knockout in female mice has been shown to exacerbate diet-induced atherosclerosis in the aorta, suggesting a protective role for androgen receptor signalling in cardiovascular health [193] (Figure 12).

### 5.6. Microbiota

In humans, distinct microbiota have been identified in various body sites, including the oral cavity, respiratory tract, skin, gut, and vagina. The gut microbiota, hosting around 100 trillion microorganisms, plays a significant role in cardiometabolic diseases, with changes linked to dyslipidaemia, dysglycaemia, arterial hypertension, and obesity [194]. In healthy adults, the gut microbiota predominantly consists of the *Bacteroidetes* and *Firmicutes* phylae. *Bacteroidetes* are crucial for polysaccharide degradation and regulation of calorie absorption [195], while *Firmicutes* contribute to the production of short-chain fatty acids, which are important for blood pressure control and glucose homeostasis [196]. *Proteobacteria*, *Actinobacteria*, *Fusobacteria*, and *Verrucomicrobia* comprise a small proportion of the bacteria in the healthy gut microbiota [197]. The balance and diversity of these microbial communities are essential for the maintenance of metabolic health and influence disease outcomes.

The *Firmicutes*/*Bacteroidetes* ratio in the gut microbiota increases from birth to adulthood and has been proposed as a marker of biological aging [198]. This ratio is associated with obesity [199], adiposity [200], and gut dysbiosis [201] and is higher in women than in men [202,203]. Additionally, the proportion of *Bacteroidetes* in the gut microbiota is generally lower in healthy women and female mice compared to their male counterparts [202,204]. However, in obesity characterized by a high BMI, the proportion of the *Bacteroides* genus is lower in men than in women [204]. Research in rodents indicates that the composition of the gut microbiota is similar in male and female mice before puberty (around 3 weeks of age) but diverges significantly after puberty (around 6 weeks of age) [205,206]. This finding suggests that sex hormones may influence gene expression and other processes in the gut microbiota, affecting microbial composition and function.

In women with PCOS, a condition characterized by hyperandrogenism and disrupted ovarian or adrenal function, there is a notable association with metabolic disorders such as obesity, IR, and T2D [207]. These metabolic disturbances are often linked to gut microbiota dysbiosis. Women with PCOS tend to exhibit higher levels of *Bacteroidetes* (including the *Bacteroidaceae*, *Porphyromonadaceae*, and *S24-7* families) and lower levels of *Firmicutes* (such as *Clostridiaceae*, *Erysipelotrichidae*, *Lachnospiraceae*, *Lactobacillaceae*, and *Ruminococcaceae*). This microbial imbalance can adversely affect the production of short-chain fatty acids, compromise gut barrier integrity, and impair immune function [208]. In rodent models, ovariectomy has been associated with decreased proportions of *Bacteroidetes* and increased *Firmicutes* compared to controls [209]. Similar findings have been observed in HFD-fed castrated mice [210], which may be related to HFD-induced abdominal obesity. These observations highlight the impact of hormonal changes and diet on the composition of the gut microbiota and its implications for metabolic health.

### 5.7. Cellular Metabolism and Mitochondria

Mitochondria play a central role in cellular metabolism. They are essential in catabolizing nutrients into ATP, generating biosynthetic precursors for macromolecules such as steroid hormones, maintaining redox homeostasis, and managing metabolic waste [211]. Energy metabolism is crucial in cardiac cell function, as the heart relies on oxidative phosphorylation for over 90% of its energy needs. Thus, mitochondrial dysfunction is a significant underlying factor in various pathologies, including metabolic disorders, renal dysfunction, and cardiovascular diseases, many of which exhibit sexual dimorphism [14].

Mitochondrial function is less efficient in males than in females. This difference is described by the Frank–Hurst hypothesis, also known as the “mother’s curse”, which suggests that alleles of mitochondrial gene accumulation may be more detrimental to males [212]. In male rodents, mitochondrial dysfunction is linked to increased susceptibility to obesity, IR, and metabolic alterations [14]. Moreover, elevated levels of methylmalonic acid, a marker of mitochondrial dysfunction, have been associated with a higher risk of future mortality in diabetic men compared to women [213]. Additionally, cardiac mitochondria have greater mitochondrial calcium retention capacity in females than in males [214]. This enhanced capacity may contribute to the observed sex-related differences in the development and progression of cardiometabolic disorders.

### 5.8. Oxidative Stress

Oxidative stress is defined as an imbalance between production of oxidative reactive species and antioxidant defences [215]. The latter are differentially expressed and produced or active according to sex. Oxidative stress plays an important role in the development of cardiovascular and metabolic disorders [216].

#### 5.8.1. Reactive Oxygen Species and Source of Production

Molecular oxygen (O_2_) possesses an oxidizing character because of its electron configuration, leading to the formation of radical and non-radical oxidants, including reactive oxygen (ROS) and nitrogen species. Primary oxidants, such as superoxide anions ($O_{2}^{-}$), hydrogen peroxide (H_2_O_2_), NO, and hydroxyl radicals (HO·), are generated physiologically and play key roles in the redox regulation of various genes. These oxidants modulate cellular functions and defence mechanisms, including pathogen destruction and tumour cell apoptosis [217].

Epidemiological and experimental studies have shown that males are more prone than females to oxidative stress, characterized by higher production of ROS and increased biomarkers of oxidative damage to proteins, lipids, and DNA [218,219]. In contrast, ROS production is often reduced in females, a phenomenon that aligns with the ’Mitochondrial Theory of Aging’ [220]. This theory suggests that the lower mitochondrial oxidant production rate in those with longer longevity, including females, contributes to their longer lifespan [221].

Sexual dimorphism in ROS production appears to be closely related to mitochondrial function. Mitochondria are the primary source of ROS, and research indicates that mitochondria in females produce higher levels of antioxidants and experience less oxidative damage than mitochondria in males [222]. NADPH oxidase, a key enzyme in ROS production, is composed of six subunits (Nox1 to Nox6) in humans [223]. Its activity is generally lower in females than males, a difference linked to oestrogen levels [217]. Among the subunits, Nox1 and Nox4 are generally expressed at higher levels in males [224], although some studies have reported higher Nox4 expression in females [225]. In isolated pig coronary arteries, expression of the Nox1 and Nox2 subunits is higher in males than in females [225]. The role of Nox2 in superoxide production has been confirmed in Nox2 knockout mice, in which a decrease in superoxide levels was observed in males but not in females [226]. However, other studies have reported no significant differences in Nox2 expression between sexes [224]. Higher expression levels of p47, a cytoplasmic subunit crucial for NADPH oxidase assembly, have been noted in males [227], although this was not influenced by oestrogen levels [217].

#### 5.8.2. Antioxidant Defences

Aerobic cells have developed complex antioxidant defence systems to counteract excessive ROS production, including enzymatic systems—such as superoxide dismutase (SOD), catalase (CAT), glutathione peroxidase (GPx), glutathione reductase (GR), thioredoxin, haem oxygenase, and paraoxonase—and non-enzymatic systems—such as glutathione; vitamins A, C, and E; uric acid; bilirubin; and albumin [217].

These defence mechanisms exhibit sexual dimorphism, with females generally showing greater antioxidant potential than males; however, this advantage diminishes after menopause due to the loss of the protective effects of oestrogens [217]. SOD activity varies by tissue, being higher in the brains, lungs, and hearts of females than those of males, although no significant difference is noted in the kidneys [228]. SOD activity decreases in both sexes following castration [229], and E2 has been shown to enhance Mn-SOD expression via the MAP kinase pathway, linking sex hormones to SOD activity [230]. Catalase activity is greater in female than in male kidneys, but no significant sex differences are observed in other tissues, suggesting less sexual dimorphism for this enzyme [228].

GPx activity also shows tissue-dependent differences: it is higher in the kidneys, brains, and livers of females compared to those of males, whereas in the heart, it is higher in males than in females [228,231]. In human erythrocytes, GPx activity is higher in adult females compared to males and is also higher in premenopausal women than postmenopausal women [232]. Moreover, in premenopausal women who have undergone total hysterectomy, GPx mRNA expression is reduced but recovers with hormone replacement therapy (HRT) [233], suggesting that oestrogens enhance GPx expression. These findings highlight the intricate relationship between sex hormones and antioxidant defence mechanisms and emphasize their role in modulating oxidative stress and influencing disease susceptibility. Women with PCOS have a poor antioxidant status, as evidenced by low vitamin E levels, suggesting that these women may experience oxidative stress, which could elevate their risk of CVD [234].

#### 5.8.3. Oxidative Stress During the Prenatal Period

In pregnant women with preeclampsia, the level of lipid peroxidation product malondialdehyde is higher than during a normal pregnancy [235]. Birth itself represents a hypoxic challenge, with significant production of free radicals. Furthermore, during parturition, several pro-inflammatory mediators, such as prostaglandin E2, arachidonic acid metabolites, and cytokines TNFα and IL-6, are produced and have been identified as important contributors to ROS production. These, in turn, lead to the production of more inflammatory signals, creating a vicious circle [236]. Bilirubin has powerful antioxidant activity and has been shown to be able to protect from 10,000-fold molar excess of hydrogen peroxide [237]. After delivery, higher levels of bilirubin are observed in mothers of girls than of boys, possibly indicating a compensatory antioxidant mechanism to combat a more oxidative environment. In addition, the placenta has been shown to transfer large amounts of bilirubin to baby girls, enhancing protection against oxidative stress. Oxytocin, higher levels of which are observed in women and during parturition [238,239], has antioxidant and anti-inflammatory properties and has been shown to modulate bilirubin levels. In addition, less oxidative damage has been observed in the mothers of girls after childbirth and in the umbilical artery of girls compared to the oxidative damage shown in the mothers of boys [240].

### 5.9. Programmed Cell Death

#### 5.9.1. Cellular Senescence

Cellular senescence is a biological response to noxious stimuli, causing cell cycle arrest and quiescence. Senescent cells adopt a flattened, enlarged morphology, accumulating insoluble proteins and lipofuscin, a high-oxidation marker [241]. This state involves reduced DNA replication, halted proliferation, and alterations in cell-cycle regulators like pRb, p21, p16^INK4a^, and p53 [242]. Senescent cells also undergo chromatin and secretome changes, genomic/epigenomic damage, unbalanced mitogenic signalling, and tumour suppressor activation [243].

Replicative senescence is irreversible, marked by telomere shortening [244], and linked to CVD and vascular pathology [245,246]. Conversely, stress-induced premature senescence (SIPS) is reversible, triggered by oxidative stress, and associated with p16^INK4a^ overexpression and reduced Sirtuin-1 functionality [247], which represents a family of proteins involved in several cellular processes, including mitochondrial biosynthesis, lipid metabolism, apoptosis, cellular stress response, fatty acid oxidation, insulin secretion, and aging [248]. The protective effect of Sirtuin-1 against cellular senescence may be exerted via the activation of endothelial NOS (eNOS), as well as by its decreasing effects on ROS production, inflammation, and DNA damage [249]. Senescent cells secrete growth factors, pro-inflammatory cytokines (IL-6, IL-1, IL-8), macrophage-inflammatory proteins, insulin-like growth factor, and extracellular matrix-degrading proteins, forming the senescence-associated secretory phenotype (SASP) [250].

Sexual dimorphism has been demonstrated in the molecular mechanisms associated with senescence: in human peripheral blood lymphocytes, repair of DNA damage has been shown to decrease more with age in women compared to men [251]. On the other hand, higher levels of senescence markers were observed in male mice compared to females, characterized by increased expression of p16^INK4a^ and p21^Cip1^ mRNA in the liver, kidneys, and spleen [252] and increased lipofuscin deposition [164]. p53 overexpression was associated with increased lifespan in males but shortened longevity in females [253]. The average length of telomeres in leucocytes is between 0.1 and 0.3 kb longer in females than in males [254]. Additionally, the rate of telomere attrition in adult leucocytes is slightly higher in males, which could predispose males to a higher risk of CVD and contribute to a shorter life expectancy. In *Mus spretus* mice, the length of telomeres is comparable to that observed in humans [255]; the leucocytes from the umbilical cord blood of female mice have longer telomeres than those from males immediately after birth [256].

Sexual dimorphism in Sirtuin-1 is not well understood, but oestrogens have been shown to modulate its functionality. In women, aging is associated with a downregulation of both Sirtuin-1 and Sirtuin-3 expression in the left ventricle, which is linked to a decline in mitochondrial antioxidant defence and an increase in inflammatory responses [257]. Ovariectomy in ApoE knockout mice resulted in reduced arterial expression of Sirtuin-1. However, administration of E2 was shown to delay senescence and the development of atherosclerotic lesions in these mice, but this effect was negated by the administration of sirtinol, a synthetic inhibitor of Sirtuin-1 [258].

#### 5.9.2. Apoptosis

Cardiac failure and aging lead to irreversible damage to cardiomyocytes, which can be eliminated through apoptosis, a form of programmed cell death [259]. Apoptosis involves specific biochemical and morphological changes, such as cell shrinkage, chromatin condensation, apoptotic body formation, and DNA fragmentation [260]. Studies have shown that aging is associated with a larger reduction in cardiomyocyte numbers in males compared to females [261]. Additionally, following myocardial injury, males have more extensive apoptosis, necrosis, and increased collagen content in the affected area, a pattern present in both mouse models and humans [262].

AMP-activated protein kinase (AMPK) has a significant role in regulating apoptosis [263], exhibiting pro- and anti-apoptotic effects. It is influenced by E2 [264] and testosterone [265], and its interaction with these hormones can help protect the cardiovascular system by reducing apoptosis [27]. The pro-apoptotic actions of AMPK are linked to the activation of caspase-3 and c-Jun N-terminal kinases (JNKs) [263], as well as the upregulation of p53, which inhibits the cell cycle in various cells, including pancreatic, liver, and vascular smooth muscle cells [266]. In neonatal rat cardiomyocytes under ischaemic conditions, AMPK activation triggers p38-MAPK-mediated translocation of the pro-apoptotic protein Bcl-2-Associated X-protein (BAX) to the mitochondria, leading to apoptosis [267]. Conversely, AMPK activation has been shown to prevent cardiomyocyte apoptosis under conditions of oxidative stress, hypoxia, ischaemia/reperfusion, cardiotoxicity, and fatty acid metabolism disorders [268,269].

Sex-related differences have been observed in the expression of several genes involved in apoptosis. In Fischer 344 rats, certain pro-apoptotic genes, including *Bad*, *Bnip3l*, *Casp3*, *Dap*, *Dapk1*, *Dapk2*, *Dapk3*, *Dffa*, *Dffb*, *Pdcd10*, *Pdcd6*, and *Vdac1*, are more highly expressed in male hearts than in female hearts [270]. This increased expression of pro-apoptotic genes in males may significantly impact cardiac function and contribute to sex-specific differences in cardiac health.

#### 5.9.3. Autophagy

Autophagy plays a crucial role in regulating various modes of cell death. It is a catabolic process that involves the degradation of cytoplasmic contents and organelles through the lysosomal pathway. This process is essential for maintaining cellular homeostasis by removing damaged or unnecessary components and ensuring cellular quality control [271]. Under physiological conditions, autophagy is constitutive and exerts housekeeping functions by regulating the integrity of intracellular compounds to limit necrosis and inflammation and to decrease ROS production. Autophagy also induces cell cycle arrest, prevents genome instability and tumorigenesis [272], and mediates a senescent cell phenotype [273]. The mammalian target of rapamycin (mTOR) is crucial. It regulates not only autophagy but also cellular processes such as growth, proliferation, motility, survival, and metabolism [274]. Downregulation or inhibition of mTOR has been linked to improved longevity in various models [275], whereas its activation can be detrimental, as observed in the hearts of aged mice [276]. mTOR functions as the catalytic subunit of two distinct protein complexes: mTOR Complex 1 (mTORC1) and mTOR Complex 2 (mTORC2) [277].

In T2D, thrombosis, hypertension, CHD, cardiac fibrosis, and aging, autophagy often diminishes, reducing its ability to confer cytoprotection [23,278,279,280]. This decline in autophagic activity impairs the cell’s capacity to remove damaged cellular components, thereby failing to prevent cardiomyocyte death and contributing to disease progression [281].

Sexual dimorphism plays a role in the regulation of autophagy across various human diseases, including CVD [174]. For example, in myocardial ischaemia/reperfusion injury, males exhibit worsened cardiac damage with decreased autophagy and increased apoptosis, whereas females show less cardiac damage, reflecting increased autophagy and reduced apoptosis [282,283]. In a mouse model of deoxycorticosterone acetate (DOCA) salt, rapamycin prevented maladaptive cardiac remodelling in males associated with a decrease in mTORC1 signalling effectors but an increase in mTORC2 signalling effectors. In females, mTORC1 and mTORC2 signalling was strongly inhibited by rapamycin, and significant downregulation of ERβ was observed [284]. In gonadectomized rats, ischaemia/reperfusion had no effect on mTOR activation in male hearts but enhanced mTOR signalling in females [283].

Other types of programmed cell death, such as necroptosis, pyroptosis, and ferroptosis, have been identified as key factors in cardiometabolic disorders [285,286,287], but as these are fairly recent areas of research, limited data on the sexual dimorphism of cardiometabolic diseases are available at the moment, which is why we do not go any further on this subject.

### 5.10. Inflammation

Inflammation is a defence mechanism of the immune system in response to harmful stimuli [288]. It proceeds by stimulating and facilitating the recruitment of immune cells to respond to the insult. Later, it triggers the healing process and restores homeostasis [289]. However, when uncontrolled, acute inflammation may become chronic and promote chronic inflammatory diseases [290]. The link between inflammation in obesity and CVD development is well known, and the presence of sex-specific activation of immune and inflammatory pathways that account for sex differences in CVD is increasingly recognized. As an example, testosterone has been shown to potentially protect against vascular aging by positively influencing vascular remodelling via a pathway associated with inflammatory cytokine release and cell survival. This sexual dimorphism is also reflected in immune pathway biomarkers, as shown by the overexpression of certain inflammatory biomarkers in females compared to males [184,291].

Collective evidence from several studies shows that there is a significant difference between men and women in the activity and efficiency of the immune system. Females tend to mount more efficient immune responses when confronted with antigenic challenges, whereas males often have a more aggressive inflammatory immune response to microbial triggers, which can have harmful effects. Certain autoimmune diseases are also more common in women than in men [292,293].

Sex hormones influence the immune response by acting directly on B and T cells, since these cells express ER and androgen receptors. Studies have shown that 17β-E2, in particular, is involved in the increased immune response following infection, as well as in the increased risk of developing autoimmune disorders. In contrast, androgens have been shown to have suppressive effects on immune functions following trauma and subsequent sepsis, with testosterone and dihydrotestosterone decreasing the production of cytokines and immunoglobulin and, thus, limiting lymphocyte proliferation [292].

#### 5.10.1. Immune Cells

Oestrogens can modulate the expression of anti- and pro-inflammatory cytokines by regulating gene expression in monocytes and macrophages [9]. For example, the *MCP-1* gene is regulated by E2. In monocytes, the production of *MCP-1*-dependent ROS has been linked to left ventricular dysfunction. Moreover, the presence of ER on immune cells appears to be necessary for regulation of inflammation in response to cardiac injuries: cardioprotective effects of E2 on neutrophil infiltration, necrosis, and oxidative stress following ischaemia/reperfusion have been reported to be dependent on ER. Finally, in ovariectomised rats subjected to ischaemia/reperfusion, administration of E2 was associated with reduced levels of TNFα in the myocardium compared to untreated rats; this was associated with improved functional recovery, as well as reductions in apoptosis and tissue injury markers [3].

Human studies also highlight distinct T-cell profiles in men and women. The proportion of circulating CD4+ T cells was found to be greater in women, who produced more interferon γ, considered in this case as an anti-inflammatory marker. In men, the smaller proportion of circulating CD4+ T cells resulted in production of more IL-17, a pro-inflammatory marker [9]. These sex-specific T-cell variations are also apparent in autoimmune disorders such as systemic lupus erythematosus (SLE). Women with SLE have altered T-cell composition, with more TH17 cells and fewer regulatory T-cells compared to women without SLE, leading to a tenfold increased risk of developing arterial hypertension compared with healthy women [9].

#### 5.10.2. Inflammasome and Cytokine Profile

The NOD-like receptor (NLR) family pyrin domain-containing 3 (NLRP3) inflammasome, a multicomponent complex, controls the maturation and secretion of IL-1β, IL-1α, and IL-18 cytokines [294] and is involved in chronic low-grade inflammation. It plays a key role in CVDs, particularly atherogenesis [295,296], and metabolic disorders [297]. In LDL receptor-deficient mice, testosterone inhibited the NLRP3 inflammasome, reducing inflammation in atherogenesis, while oestrogen promoted it [298]. A study on Saudi adults (30–65 years) found sex differences in circulating NLRP3 levels, which increased with MetS components only in women. Conversely, men without MetS had higher NLRP3 levels than women, highlighting sex-based immune differences [299].

Concerning inflammatory cytokine profiles, IL-10 inhibits pro-inflammatory cytokines and macrophage activity, while IL-6 can induce IL-10 production and regulate metabolism. In early obesity development, male and female rodents showed distinct cytokine profiles. HFD females had higher IL-6 and IL-10 levels, correlating with an anti-inflammatory profile, unlike control females or males. The IL-6/IL-10 axis may reduce inflammation in female adipose tissue, contributing to lower weight gain and better glucose tolerance than males [300].

#### 5.10.3. Other Inflammatory Markers

Adiponectin is a cardioprotective adipokine secreted by adipose tissue under several conditions, including inflammation, hypoxia, and the presence of ROS, and has been related to cardiometabolic risk. Adiponectin reduces inflammation and atherogenesis processes and improves glucose tolerance and insulin sensitivity [301,302]. Higher levels of adiponectin have been observed in women than in men in healthy populations [303,304]. In the Framingham offspring study, low adiponectin levels were identified as a significant independent risk factor for CHD only in men [302]. However, sex hormones do not appear to be involved in the regulation of adiponectin levels [305].

C-reactive protein (CRP), released by the liver in response to cytokine stimulation during the acute phase of inflammation, is a well-studied marker of inflammation whose association with CVD is well-defined [125]. Adipose tissue has been shown to enhance hepatic CRP synthesis, particularly in women [306]. Higher CRP levels (30–50%) assessed by high-sensitivity assays have been observed in women compared to men, even after adjustment for traditional cardiovascular risk factors [125,307], notably in women with MetS [308] and after heart attack [309].

### 5.11. Endothelial Dysfunction

#### 5.11.1. Endothelium and Nitric Oxide

**Figure 13:**
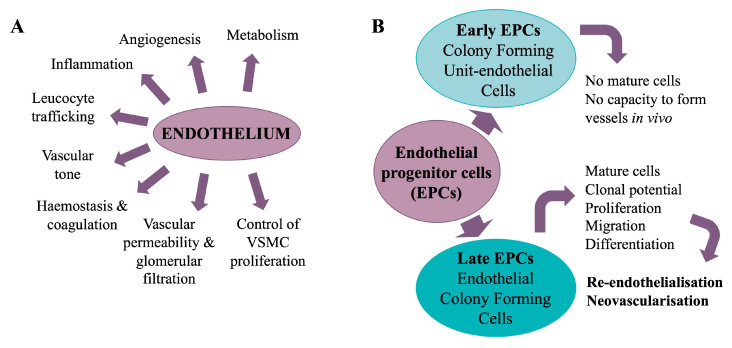
Roles of the endothelium and endothelial cell types in cardiovascular health. (**A**) Influence of the endothelium on different aspects of cardiac function. (**B**) Role of the different endothelial cell types and their characteristics. Abbreviations: VSMC, vascular smooth muscle cell; EPCs, endothelial progenitor cells.

The endothelium, a monolayer that separates the blood from interstitial fluid, plays a key role in vascular health by balancing vasodilation and vasoconstriction, regulating smooth muscle cell activity, and managing blood-clotting processes [310] (Figure 13A). NO is central to these functions, produced via eNOS from L-arginine and requiring the presence of tetrahydrobiopterin as a cofactor [164,311]. NO availability is crucial for the maintenance of vascular tone. However, NO levels decrease with age, leading to “endothelial dysfunction”, notably due to decreased eNOS expression and/or activity [216]. In addition, NO interacts with ROS, which reduces the bioavailability of NO [312] and, thus, impacts the vasodilatory response, which is essential for preserving vascular homeostasis and endothelial function [313].

#### 5.11.2. Hydrogen Sulphide

Hydrogen sulphide (H_2_S) is increasingly recognized as a key regulator of endothelial function, similar to NO. It is produced in various cardiovascular cells, including endothelial cells [314], by enzymes such as cystathionine γ-lyase, cystathionine β-synthase, and 3-mercaptopyruvate sulphur transferase (3-MST). H_2_S plays a protective role in cardiovascular health, helping to prevent CVD [315] by preserving endothelial function, stabilizing eNOS to increase NO availability, and promoting vasodilation [316,317]. H_2_S also stimulates angiogenesis by enhancing endothelial cell growth and migration [318]. In cystathionine γ-lyase knockout mice, which have low H_2_S levels, there is impaired vasodilation, hypertension, increased ROS, inflammation, and atherogenesis [317]. Conversely, supplementing H_2_S has been shown to reduce inflammation, decrease ROS, and improve vascular health through enhanced NO production [319].

#### 5.11.3. Sexual Dimorphism and Endothelial Function

Oestrogens are crucial for the regulation of endothelial function. Women generally have better endothelial function than men [313] until menopause, and men experience age-related endothelial dysfunction earlier [320,321], suggesting a role of sex hormones. Oestrogens, by binding to ERα and ERβ receptors [322], activate eNOS, leading to NO release and promoting vasodilation [323,324]. Short-term administration of ethinyl oestradiol has been shown to increase flow-mediated dilation in women with arterial hypertension [325] and reduce vasoconstriction in peri-menopausal women [326]. Oxidative stress impairs endothelial function by reducing NO production [327] and increasing vasoconstriction, partly through the activation of the renin–angiotensin system [328] by modulating the expression of Angiotensin II (AngII) type 1 and 2 receptors (AT1R and AT2R) [329]. In female rats, oestrogen deficiency results in increased vasoconstriction to AngII via upregulation of AT2R expression [330]. In addition, increased vasoconstriction to phenylephrine [331] and reduced vasodilatory effects of isoproterenol [332] have been reported. In men, oestrogens enhance vasodilation through prostaglandin I2 and NO pathways [333], whereas suppressing endogenous oestrogens in young men an in aromatase gene knockout mice impaired brachial artery flow-mediated dilation [334] and reduced endothelium-dependent vasodilation, respectively [333]. Testosterone can induce vasodilation through the relaxation of vascular smooth muscle cells (VSMCs) or by increasing NO production in an androgen receptor-dependent manner [117,335]. However, high androgen levels in women, such as in PCOS, are associated with reduced NO levels, leading to endothelial dysfunction [336].

#### 5.11.4. Endothelial Progenitor Cells

Impaired endothelial progenitor cell (EPC) function is linked to cardiometabolic disorders [216]. EPCs, key components of the endothelium, are classified by phenotype and function (Figure 13B). Early EPCs support angiogenesis via paracrine signalling but lack the ability to mature into endothelial cells. In contrast, endothelial colony-forming cells (late EPCs) exhibit clonal potential, proliferation, migration, differentiation, self-renewal, and vascular growth in vitro and in vivo [337,338]. Hill et al. [339] observed that EPCs serve as indicators of vascular reactivity, with alterations in circulating EPCs linked to cardiovascular risk factors and predictive of CVD [340,341]. Reduced circulating EPCs correlate with CVD and higher arterial BP in adults [342], while in newborns, EPC levels positively correlate with birth weight [343]. Foetal growth restriction leads to fewer functional EPCs, associated with oxidative stress and cellular senescence [344,345], persisting into adulthood in IUGR rat models [25].

In healthy middle-aged individuals, women show higher EPC colony-forming capacity and migration than men [346]. Levels of circulating EPCs are higher in premenopausal than postmenopausal women [347], with E2 positively regulating EPC mobilization via ERα [348,349]. In the absence of ERα, VEGF expression in EPCs is downregulated. However, 17β-E2 inhibits EPC migration in females but not in males [350]. EPCs also express androgen receptors; hypogonadal males have reduced circulating EPCs, restored by testosterone treatment [351]. Androgens enhance EPC vasculogenic function in vitro and ischaemia-driven angiogenesis in vivo [352].

### 5.12. Circadian Rhythm

The circadian system links the external environment to the brain and body and influences various organ functions. The central clock regulator, located in the suprachiasmatic nucleus, controls the molecular clocks throughout the body. Key transcription factors, including circadian locomotor output cycles kaput (CLOCK), play a crucial role in this regulation [353,354].

**Figure 14:**
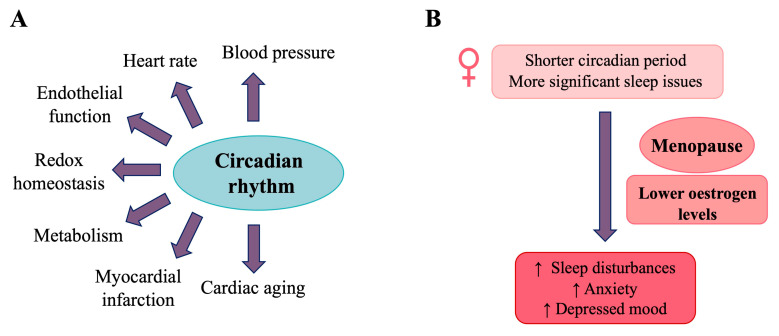
Roles of the circadian rhythm in cardiac health and specificities in women. (**A**) Cardiac functions affected by circadian rhythmicity. (**B**) Specificity of sleep issues in women aggravated by menopause.

Circadian rhythm modulates essential functions, including BP, heart rate (HR), endothelial function, metabolism, and even processes such as MI and cardiac aging [355,356] (Figure 14A). Disruptions in circadian rhythm, often due to factors like shift work (defined as three or more night shifts per month) [357], obstructive sleep apnoea [358], or social jet lag, can significantly increase the risk of CVD [359]. BP and HR naturally fluctuate with wakefulness and sleep, showing morning increases that can contribute to endothelial dysfunction, heightened MI risk [360], ventricular arrhythmias, and sudden cardiac death [361], particularly between 6 a.m. and noon [362]. Sleep disturbances are also linked to T2D and obesity [363]. The circadian clock also governs redox homeostasis [364,365]. The production of melatonin, primarily secreted by the pineal gland, is essential for the regulation of circadian rhythms, peaking around midnight [366]. Melatonin has several properties, such as anti-inflammatory, ROS-scavenging [367], antioxidant, and antiadrenergic effects, in addition to improving NO production [368]. Disrupted melatonin rhythms are evident in CVD [369], with lower serum melatonin levels in hypertensive patients [370]. Exogenous melatonin has been shown to reduce BP in both hypertensive and normotensive individuals [371,372].

Sexual dimorphism is evident in circadian rhythm disorders [373] (Figure 14B). Women are generally more sensitive to these disruptions than men [374,375], exhibiting shorter circadian periods [376] and more significant sleep issues, especially after menopause. Lower oestrogen levels contribute to sleep disturbances [377], with symptoms such as hot flashes, anxiety, and depressed mood playing a role [378]. Reduced progesterone further exacerbates irritability and relaxation difficulties [379]. Circadian rhythm disruptions heighten the risk of cardiovascular and metabolic disorders in women, leading to higher systolic and diastolic BP during acute nocturnal hypoxia [380] and elevated HR during sleep, particularly in younger women [381]. Mutations in central clock genes are linked to CVD [382], with sex differences in disease progression. For example, female CLOCK^Δ19/Δ19^ mice remain healthy longer, without cardiac dysfunction, unless they undergo ovariectomy, which suggests a protective effect of oestrogens. In contrast, male CLOCK^Δ19/Δ19^ mice rapidly develop cardiac hypertrophy and dysfunction. Aging female CLOCK^Δ19/Δ19^ mice, unlike their male counterparts, do not develop cardiomyopathy [383], further supporting the protective role of oestrogens.

### 5.13. Lifestyle

**Figure 15:**
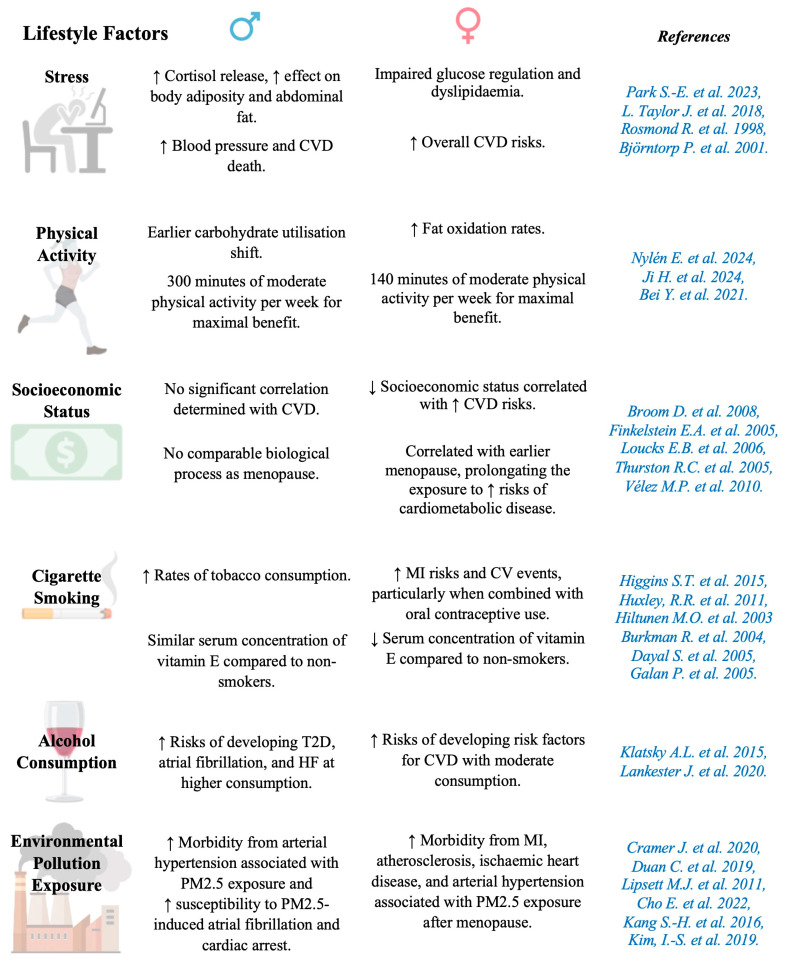
Influence of different lifestyle factors on cardiometabolic health of men and women. Lifestyle choices have different consequences for men and women. Habits and societal norms differently impact the risk of developing cardiometabolic diseases [384,385,386,387,388,389,390,391,392,393,394,395,396,397,398,399,400,401,402,403,404,405,406,407,408,409]. Abbreviations: BP, blood pressure; CV, cardiovascular; CVD, cardiovascular disease; HF, heart failure; MI, myocardial infarction; T2D, type 2 diabetes. Created using a licensed version of BioRender.com.

Various lifestyle factors significantly influence cardiovascular health. These variables include dietary habits, physical activity, stress levels, and environmental exposures. Nonetheless, the influence of these factors is not uniform among individuals. There is significant evidence of sexual dimorphism in the responses of men and women to these lifestyle factors, leading to substantial disparities in cardiovascular health outcomes (Figure 15).

#### 5.13.1. Stress

Stress is a factor that has been closely linked to an increased risk of CVD [410]. Indeed, conditions such as post-traumatic stress disorder and chronic stress have been associated with higher CVD risk. These stress-related conditions can disrupt the body’s ability to regulate its stress-response system, leading to elevated HR and BP [411]. Three main mechanisms are associated with the development of CVD under chronic stress. The first is disturbance of the hypothalamic–pituitary–adrenal (HPA) axis, increasing levels of serum cortisol, a stress hormone [411]. The second is inflammation of the arterial (endothelial) wall, contributing to the development of atherosclerosis [412]. The third is increased activity of the sympathetic nervous system, which is involved in the “fight or flight” response [413].

Men tend to be more susceptible to the effects of stress on body adiposity, BP, and CVD mortality [384]. In contrast, women are more vulnerable to the effects of stress on glucose regulation, dyslipidaemia, and overall CVD risk [385]. Existing data suggest that stress has a stronger impact on sleep and physical activity in men, whereas its effects on diet are more pronounced in women. Additionally, stress-induced cortisol release, which is greater in men than in women [386], is linked to the activation of adipose tissue and accumulation of abdominal fat [387], suggesting a potential pathway for the observed sex differences in stress-related CVD risk.

#### 5.13.2. Sedentary Lifestyle/Physical Activity

A sedentary lifestyle significantly contributes to the rise in chronic diseases like IR, obesity, high blood glucose, increased plasma lipids, and prothrombotic factors [414]. Warren et al. found that men spending over 10 h per week in a car had an 82% higher CVD mortality risk than those spending under 4 h in a car per week [415]. Similarly, Young et al. reported that lower levels of physical activity increased HF risk in 82,695 men aged 45+ [416]. Among adults aged 35–49, women were more sedentary than men, but no significant sex differences were observed in other age groups [417]. Given the health benefits of physical activity [418], interventions should address psychosocial factors influencing women’s exercise habits, including self-efficacy, social support, and motivation [419,420]. Physical activity has emerged as a potent tool in both the prevention and treatment of CVD by reducing hypertension, obesity, and dyslipidaemia [421]. The Centers for Disease Control and Prevention and the American Heart Association/American College of Cardiology recommend 150 min/week of moderate or 75 min/week of vigorous activity, plus two days of muscle strengthening [388,389,422].

Men and women have distinct physiological responses to exercise, influenced by differences in muscle mass, fat distribution, and hormonal levels, which can impact cardiovascular outcomes. For example, lower fat oxidation rates and an earlier shift to carbohydrate utilization as the dominant fuel source have been observed in men compared to women, a difference not fully explained by body fat or cardiorespiratory fitness. This variation suggests that circulating oestrogens may play a role, particularly given the association of relatively high testosterone levels in women and low levels in men with an increased risk of new-onset T2D [388]. Therefore, the effectiveness of physical activity varies considerably between sexes. For example, women achieved maximal survival benefits at 140 min/week, while men required 300 min [389]. Studies suggest that as few as 30 min of daily moderate exercise lowers CVD risk in women as effectively as longer durations in men [390].

Animal studies have revealed sex-based differences in exercise response. While both sexes showed skeletal muscle adaptations, only male rats exhibited improved heart function [390]. However, female mice demonstrated superior endurance and cardiac hypertrophy. Long-term moderate exercise reduces myocardial infarct size and prevents CAD, while short-term, low-intensity exercise aids in cardiac recovery and mimics ischaemic preconditioning [423].

#### 5.13.3. Socioeconomic Status

Socioeconomic status can shape patterns of biological and behavioural responses that have lasting effects on cardiovascular morbidity and mortality [424]. This connection is primarily influenced by factors such as poor hygiene, unhealthy lifestyle habits, and limited access to healthcare [425]. Research suggests a significant association between lower socioeconomic status and increased cardiovascular risk in women but not in men [426].

Several theories have been proposed to explain why socioeconomic status might have a stronger association with cardiovascular risk factors in women. These include differences in resource utilization [391], the greater social stigma associated with obesity for women [392], and the social and familial contexts related to lower educational attainment [393,394]. Moreover, the timing of menopause and differences in socioeconomic status may contribute to the incidence of cardiometabolic diseases. Women with lower socioeconomic status typically experience menopause earlier, leading to a longer period of exposure to increased risk [395]. In contrast, men do not experience a comparable biological process, which might reduce the influence of disparities in socioeconomic status on the incidence of cardiometabolic diseases.

Health behaviours associated with socioeconomic status also influence cardiovascular risk. Lower socioeconomic status is linked to higher rates of smoking [427], reduced physical activity [428], and problematic alcohol consumption patterns [429]. These behaviours may be driven by factors such as a poor future outlook, emotionally taxing environments, and a heightened perception of health risks in contexts of lower socioeconomic status, which can make adopting healthier behaviours more challenging [430].

#### 5.13.4. Cigarette Smoking

Cigarette smoking is a leading cause of preventable disease and disability and is responsible for about eight million deaths annually worldwide [431]. Men typically use tobacco products at higher rates than women [396], but sex differences play a crucial role in smoking patterns, related health conditions, and treatment responses, making it essential that such differences be taken into consideration in public health strategies [432].

The harmful effects of tobacco smoke are largely due to carcinogenic and mutagenic residues that generate free radicals [433,434], leading to lipid peroxidation and vascular damage [435]. Smoking alters endothelial function [436], global DNA methylation [437], and redox balance, all of which increase the risk of CVD. Notably, in women, smoking is linked to a higher risk of MI [397] and other cardiovascular events than in men [398], especially when combined with oral contraceptive use [399]. Smoking raises levels of homocysteine and asymmetric dimethylarginine (an NOS inhibitor), with both molecules increasing more in women, leading to greater endothelial dysfunction than in men [400].

Although smoking inhibits antioxidant defences [438] and downregulates Sirtuin-1 function [439], increasing ROS production in both sexes, the inflammatory response appears to be more pronounced in women. Women smokers have higher levels of platelets and monocytes [440], potentially explaining their increased risk of CHD and thrombosis [441]. Compared to female non-smokers, smoking women but not men had lower concentrations of vitamin E, which is one of the key antioxidants to protect lipids from oxidative damage [401].

Smoking and cannabis use during pregnancy are linked to complications such as placental abruption, foetal growth restriction, premature birth, and low body weight [442,443,444,445]. Long-term effects of foetal exposure to tobacco smoke include increased risks of obesity, arterial hypertension, and IGT [446,447]. Sexual dimorphism has been noted in rats, with prenatal nicotine exposure leading to a greater incidence of arterial hypertension in male offspring due to altered angiotensin receptor expression (increased AT1R expression but decreased AT2R expression) [448]. In female offspring, prenatal nicotine exposure, especially when combined with a postnatal HFD, disrupts cholesterol metabolism [449]. Female foetuses are also more susceptible to glucocorticoid overexposure, leading to lower birth weights and potential HPA-axis dysfunction [450].

#### 5.13.5. Alcohol Consumption

Heavy alcohol consumption, defined as three or more standard-sized alcoholic drinks per day, increases the risk of various CVDs, including elevated BP, stroke, irregular HR, and alcoholic cardiomyopathy [402]. However, light to moderate drinking, defined as fewer than three drinks, can provide some CV benefit, such as decreased risks of CAD, ischaemic stroke, and HF, largely due to the ability of alcohol to increase levels of HDL cholesterol and decrease blood clotting [402].

At the same or lower levels of alcohol consumption, women are more prone to develop risk factors for CVD than men [402]. Lankester et al. reported that men have higher risks of T2D, AF, and HF for every one drink more a day compared to women [403]. Overall women are less represented than men in CV studies, which influences the sex-matched alcohol dose recommendations. More research is needed to elucidate sexual dimorphism in alcohol metabolism and related CV disorders [451]. Moreover, CV benefits of low alcohol intake are possibly linked to confounding factors, such as a lifestyle and socioeconomic status [452].

Alcohol intake during pregnancy represents a major public health problem and leads to negative outcomes in newborns [453]. Prenatal alcohol exposure can lead to reduced birth weight and altered cognitive, morphological, and motor functions [454].

#### 5.13.6. Environmental Pollution Exposure

Air pollution represents a harmful mixture of solid particles and gases that can adversely impact human health on a global scale. The Global Burden of Disease 2019 study attributes 6.67 million deaths worldwide to air pollution, making it the fourth leading risk factor for global mortality [455]. This figure exceeds mortality from other modifiable cardiac risk factors such as obesity and cigarette smoking [456]. Among the components of air pollution, fine particulate matter (PM)—more specifically, particles ≤ 2.5 μm in diameter (PM2.5)—is the main substance responsible for adverse health effects [457]. PM2.5 has long-lasting biological impacts due to its high surface-to-mass ratio, which facilitates the absorption and deposition of toxic chemicals in the lungs via the respiratory tract [458].

Exposure to PM2.5 is associated with an increased risk of CVD, including CHD, arterial hypertension, cardiac arrhythmia, HF, and stroke [459,460]. In addition, the Global Burden of Disease assessment estimates that 20% of global T2D cases are linked to chronic exposure to particulate matter [461], exacerbating morbidity and mortality rates [462]. Exposure to environmental pollutants induces endothelial dysfunction [463], oxidative stress [464], platelet activation, impaired cellular signalling, epigenetic changes, and alterations in lipid and glucose metabolism. These processes contribute to a range of cardiometabolic conditions, including arterial hypertension, MI, HF, T2D, dyslipidaemia, atherosclerosis, plaque rupture, and thrombosis [465].

Sexual dimorphism has been observed in the incidence and mortality of CVD associated with exposure to PM2.5. Women, particularly in the postmenopausal period, show a stronger association between exposure to PM2.5 and increased morbidity from CVD, such as MI [404], atherosclerosis [405], and ischaemic heart disease [406], compared with men [407]. However, some studies report contrary results [466,467]. Sensitivity to air pollution exposure and its effects on different types of CVD also vary between the sexes. For instance, arterial hypertension and CHD morbidity are higher in men and postmenopausal women, suggesting a protective effect of oestrogen in premenopausal women. Conversely, men are more susceptible to PM2.5-induced AF and out-of-hospital cardiac arrest [408,409]. To explain these sex-related differences in air pollution-associated CVD, it has been suggested that women face higher levels indoor air pollution exposure due to inefficient cooking and heating with solid fuels in poorly ventilated homes [468], while men encounter more traffic-related pollution from commuting and outdoor activities [469]. Lifestyle factors like smoking and alcohol use further heighten men’s sensitivity [465]. Additionally, sexual dimorphism influences key mechanisms of air pollution-induced CVD, including inflammation, oxidative stress, endothelial damage, metabolic dysfunction, and autonomic nervous system activation [465].

## 6. Therapeutic Options

Sexual dimorphism has been identified in several aspects of oral drug absorption, including in gastric emptying time, intestinal motility, intestinal and hepatic blood flows, and bile secretion and excretion [470]. Compared to men, women have higher gastric alcohol dehydrogenase activity, which may contribute to a lower threshold for alcohol toxicity and, thus, accelerate alcohol-induced liver damage [471]. Activity of P450 enzyme CYP3A4, which is involved in the detoxification of bile acids, the termination of steroid hormone action, and the elimination of phytochemicals [472,473], is also higher in women, contributing to faster drug metabolism [474]. In addition, the higher average level of body fat in women contributes to greater volumes of distribution for lipid-soluble drugs [475] but lower volumes of distribution for water-soluble drugs [476]. Renal clearance also differs between sexes, often being lower in women [477], who have lower glomerular filtration rates than men [476]. Lower renal clearance can prolong the half-lives of drugs eliminated by the kidneys, necessitating dosage adjustments to avoid toxicity [478].

### 6.1. Adverse Drug Reactions in Women and Dosage Adaptation

In addition to responding less efficiently to therapies and treatments [8], it is well known that women are more prone to develop adverse drug reactions than men, with a 50% to 70% greater risk of adverse effects [14]. This sexual dimorphism in adverse drug effects has been described for different drug classes, including diuretics, anticoagulants, β blockers (BBs), digoxin, and angiotensin-converting enzyme inhibitors (ACEIs) [479,480]. Because plasma drug concentrations are higher in females, specifically with antithrombotic agents, the risk of bleeding complications is also significantly increased [481]. Adverse drug reactions in women are also more serious than in men. For example, 60% of patients hospitalized because of adverse effects of drugs are women.

Women are also more likely to be treated more conservatively. Indeed, women are often treated with the same pharmacological therapies as men; however, studies have shown that women with ACS are less likely to benefit from reperfusion therapies, coronary artery bypass grafting, or coronary angiography compared to men. This disparity in treatment response has been associated with higher in-hospital mortality rates for women [26]. Risk factors for adverse drug events may, in part, be related to polytherapy, as older age and depression are more frequent in women [14]. Despite recognition of these facts, specific pharmacological protocols adapted to patient sex are still lacking, with identical guidelines and doses for men and women. Women are also under-represented in clinical trials, so treatments and drugs are often developed and tailored specifically for men rather than being optimized for women. This lack of sex-specific research leads to disparities in treatment efficacy and outcomes, highlighting the need for more inclusive clinical studies that consider the physiological and hormonal differences between sexes.

### 6.2. Antihypertensive Treatment

Several studies have shown that women are frequently treated with loop diuretics, thiazide diuretics, aldosterone-receptor blockers, and BBs but less frequently than men with ACEIs, AngII receptor blockers (ARBs), and calcium channel blockers (CCBs) after adjustment for various confounding factors [482]. Other studies have observed greater ARB use in women than in men [483,484]. BBs have been shown to improve survival only in hypertensive men, not in women [485]. However, the use of combined therapy with diuretics and CCB increased CVD mortality (85%) in women compared with those treated with diuretics and BBs [486]. In terms of CVD prevention, treatment with CCBs has been shown to reduce the risk of stroke more effectively in women than in men [487]. However, CCBs were less effective than BBs and diuretics in preventing HF in both sexes. Additionally, CCBs were inferior to ACEIs only in men.

### 6.3. Cardiovascular Disease Treatments

#### 6.3.1. Aspirin

Aspirin is an antithrombotic agent that inhibits the synthesis of thromboxane A2, a potent platelet aggregator, resulting in positive effects for CVD prevention [488]. Although aspirin treatment reduces the risk of non-fatal MI in men, it appears to be less effective in women [489].

The known structural and physiological differences in coronary vascularization between men and women [490] have led to the hypothesis that women may be more resistant to aspirin therapy [491,492]. Women typically have smaller and stiffer coronary vessels than men, which may promote fibrotic tissue deposition and vessel wall remodelling. Additionally, women are more likely to exhibit impaired vasodilatory responses to acetylcholine [493], and their atherosclerotic lesions tend to be more diffuse compared to those in men [494]. In a rabbit model, aspirin has been shown to reduce the *thrombus* size only in males [495], which aligns with human studies indicating that only men experienced a reduction in *thrombus* size with aspirin [496]. This observed sexual dimorphism may arise from differences in underlying mechanisms, including in the fibrinolytic system, platelet prostaglandin synthesis, and the interaction between platelets and the vascular wall in response to the salicylate fraction of acetylsalicylic acid. Furthermore, platelets in men aggregate at lower concentrations of aggregating agents than platelets in women, suggesting differing sensitivities to these agents [497]. Therefore, clinicians should exercise caution when prescribing aspirin to women, particularly for primary prevention [489].

#### 6.3.2. Statins

Statins are lipid-lowering therapies that have been shown to improve endothelial function and to be anti-inflammatory [498]. The beneficial effects of statins are the result of their capacity to reduce cholesterol biosynthesis, mainly in the liver, where they are selectively distributed, as well as to influence lipid metabolism, as a result of their inhibitory effect on 3-hydroxy-3-methyglutaryl coenzyme A (HMG-CoA) reductase [499].

A 2022 study by Hunt et al. reported a significantly greater increase in HDL cholesterol in women than men after initiating statin therapy [500]. These findings contrast with a meta-analysis by Karlson et al., in which a greater increase in HDL cholesterol was reported for men receiving statins and greater decreases in LDL cholesterol were reported for women [501]. In a cohort study conducted using the United Kingdom Biobank, regular statin use was associated with a 23% decreased risk of irritable bowel syndrome among male participants, but no significant association was found in female participants [502]. Additionally, Nanna et al. highlighted sex differences in the use of statins in community practice, revealing that women were more likely to report never being offered statin therapy, as well as more likely to decline or discontinue the treatment [503].

#### 6.3.3. Digitalis

Digitalis is a plant that produces its effects primarily by modulating the autonomic nervous system. However, at toxic concentrations, stimulation of the sympathetic nerve can also occur, potentially leading to arrhythmia [504]. Digoxin is one of the oldest drugs used to treat heart problems such as HF, AF, and atrial flutter, notably by increasing BP, myocardial contractility, and stroke volume. In HF and depressed left ventricular systolic function, digoxin therapy has been associated with an increased risk of death from any cause among women but not men [505], raising concerns about the safety of digoxin treatment in females [506].

### 6.4. Oxidative Stress Management

The development of therapies to limit oxidative stress may represent an interesting approach to prevent cardiometabolic disorders, as adequate total antioxidant capacity levels in the body prevent the development of CVD.

#### 6.4.1. Antioxidant Compounds

In animal models of arterial hypertension, treatment with antioxidants has been used successfully. Oral treatment with lazaroid, an ROS scavenger, improved NO viability and reduced arterial BP in spontaneously hypertensive rats [507], as well as in a rat model of IUGR [508]. Similar results have been reported in rats using treatment with N-acetylcysteine, an inhibitor of ROS production [509], and with allopurinol, a xanthine oxidase inhibitor [510].

In contrast to results from preclinical models, clinical trials of antioxidant strategies for the treatment of arterial hypertension were not successful. This complexity likely stems from the diverse nature of the condition. Although all patients with BP values above a certain threshold are classified as hypertensive [511,512], this broad definition encompasses a wide variety of phenotypes, including young, lean individuals; the obese; postmenopausal women; and the elderly. Each phenotype is associated with different aetiologies influenced by various risk factors (such as genetics and family history), lifestyle (including smoking, diet, and physical inactivity), and concomitant conditions (like chronic kidney disease and T2D). Consequently, the role of oxidants may vary across these phenotypes and might be obscured in clinical trials that do not appropriately select patient subgroups.

#### 6.4.2. Vitamin D

Vitamin D deficiency is common in Western populations and linked to higher risks of CVD [513,514]. Low vitamin D levels may increase renin and AngII production, leading to arterial hypertension, left ventricular hypertrophy, inflammation, and atherosclerosis. Additionally, deficiency contributes to IR and pancreatic β-cell dysfunction, increasing the risk of MetS and T2D [515,516]. Vitamin D acts as an antioxidant by preventing lipid peroxidation in the cell membrane [517], in addition to reducing hydrogen superoxide-induced oxidative stress and preventing ROS production by inhibiting MEK/ERK/Sirtuin-1 axis switching [518,519]. Vitamin D also exerts an anti-inflammatory and anti-fibrotic effects [520]. Its effects depend on intake and interaction with the vitamin D receptor (VDR) [521,522], which is present in multiple organs, such as the gut, skeleton, parathyroid gland, ovaries, and testicles. Vitamin D influences oestrogen biosynthesis by regulating calcium homeostasis and the aromatase gene (CYP19A1), which impacts fat distribution and lipid metabolism [522]. The CYP19A1 rs10046 polymorphism is linked to higher cardiovascular risk due to its role in increasing atherogenic lipoproteins and contributing to IR, T2D, and arterial hypertension [521].

Women, especially those under 20 and over 80, are more prone to vitamin D deficiency [523], while overweight/obese men are also at risk [524,525]. In women, low vitamin D is associated with more severe CAD [526] and stroke risk due to cerebral artery vasoconstriction [527]. In men, vitamin D deficiency correlates with elevated alanine aminotransferase levels, cholesterol, and triglycerides [528].

#### 6.4.3. Dietary Antioxidant Capacity

The dietary antioxidant capacity reflects all the antioxidant compounds present in food and the interactions between those compounds [522]. The total dietary antioxidant capacity is inversely associated with CVDs such as HF [529], MI [530], and stroke [531]. A higher dietary antioxidant capacity has been linked to a lower prevalence of arterial hypertension, reduced haematocrit and total cholesterol, and increased albumin and vitamin D concentrations, particularly in men. As a result, this improved antioxidant intake may offer protection against cardiometabolic risk factors in this population [532]. Antioxidant vitamins and minerals, such as vitamins A, E, and C and zinc [533], have been associated with a decline in the development and progression of CVD [534].

Few data on the differential effects of these antioxidant therapies in men and women have been published; however, one study reported that there were no overall effects of vitamins C, E, or beta-carotene on cardiovascular events among women at high risk for CVD [535]. Similarly, ascorbic acid had no effect in terms of preventing coronary disease in either sex [536]. In one study, a protective action of ascorbic acid on peripheral arterial disease was present in women but not in men [537]. In male smokers, vitamin E supplementation increased erythrocyte SOD activity and decreased GPx activity, improving oxidative stress control [538].

In animal studies, supplementary antioxidant therapy (vitamin E, beta-carotene, and vitamin C) in LDL receptor-null female mice fed a high-fat, high-cholesterol diet had beneficial effects in terms of reducing LDL oxidation and fatty streak lesion development [539]. In rabbits fed a cholesterol-rich diet, alpha lipoic acid supplementation had dual lipid-lowering and anti-atherosclerotic effects, characterized by low total cholesterol and LDL plasma levels and a reduction in athero-lesion formation in hypercholesterolaemically induced male rabbits [540].

The American Heart Association recommends that vitamin and mineral supplements only be taken in addition to a healthy eating pattern and only with the recommendation of a physician or dietitian; however, it recommends eating foods rich in antioxidant vitamins, especially fruit and vegetables. While it is not clear which dietary antioxidants are responsible for the cardiovascular risk reduction, considering all the studied antioxidants, the Mediterranean diet currently seems to offer the best outcomes [541].

### 6.5. Nutrition

#### 6.5.1. Mediterranean Diet

The Mediterranean diet is now widely recognized for its health benefits, including reduced risks of cardiovascular and metabolic diseases [541]. This diet is characterized by a high consumption of plant foods (fruit and vegetables) and whole grains (cereals, bread, rice, or pasta, and nuts containing antioxidants); moderate intake of dairy products (cheese and yogurt) and fatty fish rich in polyunsaturated fatty acids; low consumption of red meat, processed meats, and sweets; and consumption of zero to four eggs per week. Extra virgin olive oil is the main source of fats, and wine is consumed in small to moderate quantities [541]. Women have been shown to adhere much more closely to the Mediterranean diet than men [542]. In addition, women, in general, eat more vegetables and fruit and, therefore, absorb greater quantities of antioxidants. They also consume more fish/seafood and nuts but less olive oil, butter, cream, margarine, red/processed meats, soft drinks, red wine, and commercial sweets [543]. This healthier diet may explain why women have lower high-sensitivity (hs) CRP levels than men. Women who developed AF were shown to be older, more overweight, and exhibit lower adherence to the Mediterranean diet [542]. However, adherence to the Mediterranean diet also had beneficial effects in men: a decrease in adiponectin concentration and a better redistribution of LDL subclasses, from smaller to larger, were observed only in men [544]. In older, overweight/obese individuals (aged around 66 years) with MetS, reductions in weight, waist circumference, fasting blood glucose, insulin, and TG levels with a Mediterranean diet were more pronounced in men than in women [545].

The Mediterranean diet has been combined with the Dietary Approaches to Stop Hypertension (DASH) diet, which was initially studied for cognitive performance [546] but that has recently been investigated for its ability to protect against cardiometabolic diseases and their risk factors, such as obesity, inflammation, and dyslipidaemia [547,548]. This combined diet (MIND) recommends eating a lot of fruits—berries in particular [549]. Research exploring the effects of the MIND diet on cardiometabolic disease has revealed sex-specific outcomes: in women, the MIND diet was negatively associated with obesity, indicating that adherence to this diet may reduce the risk of obesity specifically in women [547]. Another study showed that the serum concentration of CRP was lower in men following adherence to the MIND diet [550].

#### 6.5.2. Vegetarian Diet

Blood samples taken from age-matched healthy vegetarians and non-vegetarians revealed a purportedly beneficial increase in adiponectin levels in vegetarian women compared to men [551]. Compared with an omnivorous diet, a vegetarian dietary pattern was associated with a reduction in CVD in men, whereas in women, this relationship was less marked or non-existent [552]. In addition, low vegetable consumption was associated with the risk of T2D in men but not in women [553].

#### 6.5.3. Caloric Restriction

CR was first suggested in the early 20th century as a method of increasing lifespan and, today, remains the most reliable non-genetic intervention for delaying ageing in a variety of species [554]. CR involves reducing caloric intake by 20–40% compared to ad libitum consumption, while ensuring adequate protein and micronutrient intake to prevent malnutrition [555].

In humans, short-term CR diets generally last from 1 month to 1 year, while long-term CR can last from 3 to 15 years, with an average duration of around 7 years. Caloric intake is reduced by avoiding energy-dense foods (e.g., refined carbohydrates, potatoes, white bread, white rice, sweets, and sugary drinks) and increasing consumption of nutrient-dense foods (e.g., vegetables, fruits, nuts, low-fat dairy products, egg whites, wheat and soy proteins, fish, and lean meats) [556]. Long-term CR has, in particular, been shown to exert a protective effect against atherosclerosis (approximately 40% reduction in carotid artery intima-media thickness) and to reduce arterial hypertension (lower systolic and diastolic BP) and inflammation (lower plasma levels of CRP, TNFα, and IL-6) [557]. In addition, long-term CR was reported to significantly improve left ventricular diastolic function, reduce arterial stiffness, and improve arterial endothelial function and cardiac autonomic function [558]. Finally, long-term CR has a protective role with respect to overweight/obesity, T2D, and inflammation [556,557] and reduces levels of total cholesterol, LDL cholesterol, TG, fasting blood glucose, and plasma insulin [559].

Sexual dimorphism in the effects of CR has mainly been studied in preclinical models, especially in males [2], with some studies identifying greater CR-induced fat mass loss in males than in females [560]. In terms of longevity, female mice were found to have a greater response to CR than male mice [561], whereas the opposite was observed in rats [562]. In a study of 40% CR in rats, blood coagulation was examined in the context of CVD. The study found similar reductions in vitamin K concentrations in the two sexes under CR. However, a reduced prothrombin time was observed in males but not in females, suggesting that males had a more robust response to CR than females [563].

### 6.6. Cell Therapies

#### 6.6.1. Mesenchymal Stem Cells

The development of “super stem cells” with enhanced self-renewal and differentiation offers a promising approach for CVD treatment [564]. Mesenchymal stem cells (MSCs) can develop into cardiomyocytes and endothelial cells [565], but their clinical application is hindered by poor survival after transplantation. Ensuring their viability is crucial for effective therapy [346].

Sexual dimorphism influences MSC-based treatments, with female MSCs exhibiting greater resistance to injury due to oestrogen [565] by stabilizing mitochondrial function, and activates key survival pathways (PI3K/AKT and ERK1/2), enhancing angiogenesis, reducing inflammation, and improving cardiac repair [566].

#### 6.6.2. Induced Pluripotent Stem Cell Regenerative Therapy

Induced pluripotent stem cell (iPSC) regenerative therapy has emerged as a ground-breaking approach to treat CVD, offering the potential to generate patient-specific cardiomyocytes and vascular cells. iPSCs, derived from reprogrammed adult somatic cells, can differentiate into any cell type required for cardiac repair and regeneration. This technology hold promise to repair damaged heart tissue; improve heart function; and, potentially, cure heart disease [124].

Sexual dimorphism may also influence the efficacy of iPSC-based therapies for CVD. Oestrogens play a crucial role in enhancing the survival, proliferation, and differentiation of iPSCs into cardiomyocytes. Women, due to their higher levels of oestrogens, tend to benefit more from iPSC therapies, as the hormone offers protective effects against apoptosis and promotes cardiac cell regeneration [567], whereas lower oestrogen levels in men correlate with a weaker regenerative response [568,569].

### 6.7. Hormone Replacement Therapy

HRT is a medical treatment in which hormones, typically oestrogens and progesterone, are prescribed to alleviate symptoms associated with menopause in women [570]. Early observational studies suggested that HRT could offer significant benefits, such as a reduced risk of CHD and lower mortality rates. However, subsequent trials, which primarily involved postmenopausal women in their sixties with pre-existing health conditions, painted a more complex picture [571]. These studies identified increased risks of CHD and breast cancer, particularly within the first 1–2 years of HRT use, leading to a decline in its use among this demographic. In contrast, for healthy women aged 50 to 60 years [572], the risk–benefit profile of HRT is more favourable. In this group, HRT not only alleviates menopausal symptoms but may also reduce the risk of osteoporosis. The decision to use HRT should be individualized, considering a woman’s health status, medical history, and specific menopausal symptoms. Further reanalysis of data focusing on women within 10 years of menopause onset revealed that HRT may reduce CAD and all-cause mortality in this group. Meta-analyses have supported these findings, showing a decreased incidence of CVD with early HRT initiation. These results highlight the importance of the timing of HRT initiation relative to menopause onset in determining its benefits and risks [571,573].

Outcomes are less clear with the use of androgen therapy. The use of testosterone therapy has produced contradictory results, with studies showing effects ranging from protective to potentially harmful. This raises questions about the true benefits of androgen therapy and its long-term cardiovascular risks [335].

## 7. Conclusions

In conclusion, sexual dimorphism plays a critical role in the pathophysiology, prevalence, and progression of cardiometabolic diseases. The distinct differences between men and women evident in cardiovascular health, MetS, and the developmental origins of these disorders highlight the necessity of sex-specific approaches in research and clinical practice. Several factors contribute to the divergent patterns observed in men and women.

Understanding these variations is essential in the development of targeted therapeutic strategies such as HRT, personalized drug dosages, and lifestyle modifications tailored to each sex. Emerging therapies, including oxidative stress management, stem cell regenerative therapy, and dietary interventions, offer promising avenues to address these sex-specific disparities in cardiometabolic health.

As research continues to unveil the complex interplay between genetics, environment, and sex in cardiometabolic diseases, it is becoming increasingly clear that a one-size-fits-all treatment approach is insufficient. Future studies should focus on further elucidating these mechanisms to enhance personalized medicine, ultimately improving outcomes for men and women affected by these conditions.

## Data Availability

Not applicable.

## Glossary

ACEI, Angiotensin-Converting Enzyme Inhibitor; ACS, Acute Coronary Syndrome; AMPK, AMP-activated Protein Kinase; AngII, Angiotensin II; ApoE, Apolipoprotein E; AR, Androgen Receptor; ARB, Angiotensin Receptor Blocker; AT1/2R, Angiotensin II type 1/2 Receptor; BBs, Beta Blockers; BMI, Body Mass Index; BP, Blood Pressure; CAD, Coronary Artery Disease; CAT, Catalase; CCB, Calcium Channel Blocker; CHD, Coronary Heart Disease; CLOCK, Circadian Locomotor Output Cycles Kaput; CO, Carbon monoxide; COL(1A1), Collagen I; CR, Caloric Restriction; CRP, C-Reactive Protein; CVD, Cardiovascular Disease; DASH, Dietary Approaches to Stop Hypertension; DOCA, Deoxycorticosterone Acetate; DOHaD, Developmental Origins of Health and Disease; E2Oestradiol, eNOS, Endothelial Nitric Oxide Synthase; EPCs, Endothelial Progenitor Cells; ERK, Extracellular signal-Regulated Kinase; ER, Oestrogen Receptor; FFA, Free Fatty Acid; GA, Gestational Age; GDM, Gestational Diabetes *Mellitus*; GPER1, G Protein-Coupled Receptor 1; GPx, Glutathione Peroxidase; GR, Glutathione Reductase; H_2_O_2_, Hydrogen Peroxide; H_2_S, Hydrogen Sulphide; H3K27me3, Histone H3 Lysine 27 trimethylation; HF, Heart Failure; HDL, High-Density Lipoprotein; HDL-C, HDL-Cholesterol; HFD, High-Fat Diet; HMG-CoAm, Hydroxymethylglutaryl-CoA; HO, Hydroxyl Radical; HPA, Hypothalamic–Pituitary–Adrenal; HR, Heart Rate; HRT, Hormone Replacement Therapy; iPSC, Induced Pluripotent Stem Cell; IFG, Impaired Fasting Glucose; IGT, Impaired Glucose Tolerance; IL, Interleukin; IR, Insulin Resistance; IUGR, Intrauterine Growth Restriction; JNK, c-Jun N-terminal Kinase; LDL, Low-Density Lipoprotein; LDL-C, LDL Cholesterol; LGA, Large for Gestational Age; lncRNA, Long non-coding RNA; MAFLD, Metabolic Dysfunction-Associated Fatty Liver Disease; MAPK, Mitogen-Activated Protein Kinase; MCP1, Monocyte Chemoattractant Protein 1; MetS, Metabolic Syndrome; MI, Myocardial Infarction; MIND, Mediterranean-DASH Intervention for Neurodegenerative Delay; MiRNA, Micro RNA; MMP-2, Matrix Metalloproteinase 2; MSC, Mesenchymal Stem Cell; mTOR, Mammalian Target of Rapamycin; NADPH, Nicotinamide Adenine Dinucleotide Phosphate; NAFLD, Non-Alcoholic Fatty Liver Disease; NASH, Non-Alcoholic Steatohepatitis; NLR, NOD-Like Receptor; NLRP3, NLR family Pyrin domain-containing 3; NO, Nitric Oxide; NOX, NADPH Oxidase; OSA, Obstructive Sleep Apnoea; PCOS, Polycystic Ovary Syndrome; PI3K, Phosphoinositide 3-Kinase; PM, Particulate Matter; PM2.5, Particulate Matter ≤ 2.5 μm in diameter; RAS, Renin Angiotensin System; ROS, Reactive Oxidative Species; SASP, Senescence-Associated Secretory Phenotype; SAT, Subcutaneous Adipose Tissue; SIPS, Stress-Induced Premature Senescence; SLE, Systemic Lupus Erythematosus; SOD, Superoxide Dismutase; T2D, Type 2 Diabetes; TG, Triglyceride; TNFα, Tumour Necrosis Factor Alpha; VAT, Visceral Adipose Tissue; VDR, Vitamin D nuclear Receptor; VEGF, Vascular Endothelial Growth Factor; VLDL, Very Low-Density Lipoprotein; VSMC, Vascular Smooth Muscle Cell; WHO, World Health Organization; XIAP, X-linked Inhibitor of Apoptosis Protein; XIST, X-Inactive Specific Transcript.
